# Automated patch-clamp recordings for detecting activators and inhibitors of the epithelial sodium channel (ENaC)

**DOI:** 10.1007/s00424-025-03087-3

**Published:** 2025-05-08

**Authors:** Florian Sure, Markus Rapedius, Alexei Diakov, Marko Bertog, Alison Obergrussberger, Niels Fertig, Christoph Korbmacher, Alexandr V. Ilyaskin

**Affiliations:** 1 Institute of Cellular and Molecular Physiology, Friedrich-Alexander-Universität Erlangen-Nürnberg, Erlangen, Germany; 2 Nanion Technologies GmbH, Munich, Germany

**Keywords:** Epithelial sodium channel (ENaC), Automated patch-clamp (APC), Electrophysiology, Ion channel, Activator, Inhibitor

## Abstract

The epithelial sodium channel (ENaC) is crucial for sodium absorption in several epithelial tissues including lung and kidney. Its involvement in various renal and pulmonary disorders makes ENaC a potential drug target. High-throughput screening using the automated patch-clamp (APC) technique appears to be a promising approach to discover novel ENaC modulators with (patho-)physiological and therapeutic implications. The aim of this methodological study was to establish APC measurements of ENaC-mediated currents. First, we confirmed functional expression of ENaC in a HEK293 cell line stably transfected with human αβγ-ENaC using conventional manual whole-cell patch-clamp recordings. For APC measurements, a standard enzymatic cell-detachment procedure was used to prepare single cell suspensions. This resulted in a high success rate of APC recordings with amiloride inhibitable ENaC currents. Using a γ-inhibitory peptide and the small molecule ENaC activator S3969, we demonstrate that APC recordings could reveal inhibitory as well as stimulatory effects on ENaC. Interestingly, the enzymatic cell-detachment protocol resulted in partial proteolytic ENaC activation. The portion of proteolytically activated channels could be reduced by prolonged incubation of suspended cells in cell culture medium. This recovery protocol enhanced the relative stimulatory effect of chymotrypsin, a prototypical serine protease known to cause proteolytic ENaC activation. Thus, this protocol may be particularly useful for identifying novel ENaC activators mimicking proteolytic channel activation. In conclusion, we have established a high-throughput screening method for the identification of novel ENaC activators and inhibitors using APC.

**Supplementary Information:**

The online version contains supplementary material available at 10.1007/s00424-025-03087-3.

## Introduction

The epithelial sodium channel (ENaC) is a heterotrimeric ion channel which typically consists of an α, β, and γ-subunit and belongs to the ENaC/degenerin family of ion channels [27]. ENaC is essential for sodium absorption in several epithelia. In the distal nephron, ENaC plays a pivotal role in adjusting renal sodium excretion to sodium intake and, hence, in maintaining sodium homeostasis, extracellular volume, and blood pressure [50, 55]. Hyperaldosteronism or gain-of-function mutations of ENaC cause salt-sensitive hypertension, highlighting ENaC’s critical role in blood pressure regulation [17, 42, 61]. In addition to aldosterone, several other hormonal and local mediators regulate ENaC activity in a complex manner [26, 56]. Thus, it is plausible that subtle disturbances of ENaC’s regulatory network may lead to channel hyperactivity, thereby contributing to the pathophysiology of essential hypertension [38]. On the other hand, hypoaldosteronism and loss-of-function mutations of ENaC, like in pseudohypoaldosteronism (PHA1B), result in hypotension and renal salt wasting [5, 14]. Therefore, pharmacological modulation of ENaC in the kidney represents a promising therapeutic strategy for long-term blood pressure control. Furthermore, modulation of ENaC activity is discussed as a potential treatment option to improve pulmonary fluid balance [11] in the context of cystic fibrosis [3, 6, 7, 32], respiratory distress, and pulmonary edema [12, 18, 66].

Collectively, previous studies highlighted the therapeutic potential of ENaC activators and inhibitors. However, only a few ENaC modulators have been developed to date. The best-known ENaC inhibitor, amiloride, is used clinically as a potassium-sparing diuretic to treat hypertension [58]. Other clinically relevant ENaC inhibitors are benzamil and triamterene [38, 58]. Noteworthy is that all of these substances show a high structural similarity and probably act via the same molecular mechanism, i.e., by blocking the channel’s pore. Furthermore, their specificity towards ENaC is rather low [2, 66], and their use to target ENaC in the lung is hampered by pharmacokinetic limitations and possible renal side effects in case the drugs are absorbed into the circulation [6, 7]. Therefore, there is a need for new ENaC inhibitors with increased specificity, based on new molecular scaffolds and suitable for different modes of application (e.g., by inhalation).

In this context, it is worth noting that ENaC activation involves complex proteolytic processing of the channel. ENaC cleavage at specific sites results in the release of inhibitory tracts (autoinhibitory peptides) from the respective binding pockets localized in the extracellular domains of α- and γ-ENaC which leads to proteolytic channel activation, a unique feature of ENaC [43, 44, 56]. Consistent with this, applying synthetic peptides corresponding to α- or γ-autoinhibitory peptides (α-8: 184-LPHPLQRL-191; γ-11: 153-RFSHRIPLLIF-163) was shown to reduce ENaC activity [25, 49]. Therefore, it seems plausible that novel ENaC inhibitors mimicking the autoinhibitory peptides can be designed in future studies.

There are only few ENaC activators available to date. A well-studied ENaC activator is AP301 (Solnatide), a peptidomimetic drug that resembles the lectin-like domain of the cytokine tumor necrosis factor (TNF). AP301 is believed to stimulate ENaC through direct interaction with its α-subunit [35, 36, 60] and probably can be used as a drug to improve ENaC function in lung disorders [18, 66, 66]. In addition, a small molecule ENaC activator called S3969 has been described previously [34]. S3969 is a peptidomimetic substance that stimulated not only wildtype human ENaC but also channels carrying a PHA1B mutation or partial loss-of-function mutations associated with atypical cystic fibrosis [21, 34]. Importantly, we have recently identified the functionally relevant S3969 binding site in β-ENaC using a structure-guided approach [65]. This information can potentially be used for identification of novel ENaC activators.

To summarize, ENaC is a promising drug target but new methodological approaches are needed to identify novel ENaC modulators. Recent advances in resolving ENaC structures using cryo-electron microscopy (cryo-EM) may facilitate the development of new ENaC-targeting compounds [19, 43, 44]. In addition, the search for ENaC modulators is likely to benefit from the development of suitable high-throughput screening strategies.

The patch-clamp technique remains the gold standard for evaluating ENaC function and regulation [9, 10, 33, 39–42, 63]. However, manual patch-clamp experiments are very time- and labor-consuming, which results in low throughput hampering its use for drug discovery and other screening studies. As an alternative, the automated patch-clamp (APC) technique has been increasingly used to study ion channels since its development in the late 1990s and early 2000s. Importantly, specialized high-throughput APC platforms have been developed recently (e.g., SyncroPatch 384; [45]), which demonstrate some technical advantages over commonly used fluorometric imaging plate reader (FLIPR) screening technologies [46]. Furthermore, APC approaches are suitable for various physiological applications using different cell systems including primary cultures and stem-cell derived cell lines [48]. We are not aware of any studies demonstrating the feasibility of APC recordings of ENaC currents. Therefore, the aim of the present study was to establish high-throughput APC measurements of ENaC currents using a high-throughput 384-well APC system (SyncroPatch 384) and a commercially available HEK293 cell line with constitutive expression of human αβγ-ENaC. Furthermore, using known modulators of ENaC function, we wanted to verify that this approach enabled reliable detection of stimulatory and inhibitory effects of applied substances on ENaC currents.

## Methods

### Chemicals

Chymotrypsin and amiloride were obtained from Sigma-Aldrich and directly dissolved in the respective bath solution. S3969 was synthesized essentially as described previously [21, 34]. The ENaC inhibitory peptide (γ-11; Acetyl-RFSHRIPLLIF-Amide; [49]) was synthesized by ThermoFisher. Bath solutions containing S3969 or γ-11 were prepared from 100 mM stock solution in DMSO on the day of the experiment. Enzymatic cell detachment reagent TrypLE Express was obtained from ThermoFisher. Trypsin–EDTA (0.5%) was obtained from Sigma-Aldrich. EDTA, Collagenase, and Dispase II from Sigma-Aldrich were prepared in sterile-filtered divalent cation-free Dulbecco’s Phosphate Buffered Saline (Sigma-Aldrich) at a concentration of 1 mM, 50 U/ml, and 2.5 U/ml, respectively. Sodium citrate solution was prepared by dissolving KCl and Na-Citrate in sterile-filtered distilled water (in mM: 135 KCl, 15 mM Na-Citrate).

### ENaC-HEK293 cell culture

A stably transfected HEK293 cell line, expressing human α-, β-, and γ-ENaC subunits, was obtained from Charles River (Catalog Number CT6259). Cells were maintained in a 5% CO_2_ atmosphere at 37 °C in DMEM/GlutaMAX medium (ThermoFisher) supplemented with fetal bovine serum (10%, Cytiva Life Sciences), penicillin (100 U/ml, Sigma-Aldrich), streptomycin (100 µg/ml, Sigma-Aldrich), and selection antibiotics Hygromycin B (0.02 mg/ml, ThermoFisher), Zeocin (0.1 mg/ml, ThermoFisher), and Geneticin (0.5 mg/ml, Sigma-Aldrich), according to manufacturer’s protocol. Additionally, amiloride (50 µM) was added to the culture medium to prevent cell sodium overload due to ENaC activity. Control HEK293 without ENaC-expression were obtained from ATCC (Catalog Number CRL-1573) and cultured under the same experimental conditions, but in the absence of the selection antibiotics.

### Cell surface biotinylation and immunoblotting in HEK293 cells

Whole-cell lysates of HEK293 cells were obtained by scraping the cells from a cell culture dish and dissolving them in Lysis Buffer (composition: 50 mM HEPES, 150 mM NaCl; 1% Triton X-100, 10% Glycerol; pH 7.4) supplemented with Protease Inhibitor Cocktail (cOmplete™ Protease Inhibitor Cocktail, Sigma-Aldrich). After sonication, samples were kept at 4 °C for 1 h before centrifugation at 1,000*g* for 10 min to remove cell debris. Cell surface fractions were obtained using an established biotinylation approach [63, 64]. Cells were incubated for 30 min with 0.5 mg/ml sulfo-NHS-SS-Biotin dissolved in phosphate-buffered saline (PBS; composition in mM: 137 NaCl, 2.7 KCl, 4.3 Na_2_HPO_4_, 1.5 KH_2_PO_4_; pH 7.3) complemented with 0.5 mM CaCl_2_ and 0.7 mM MgCl_2_ (PBS-CM). This was followed by a 30 min incubation with 100 mM glycine in PBS-CM to quench remaining biotin. Cells were scraped and lysed as described above. After removing cell debris by centrifugation at 1,000*g* for 10 min, Neutravidin Beads (Pierce) were added to the supernatant for an overnight incubation to extract biotinylated proteins. Finally, the fraction of cell surface protein was separated from beads using a reducing agent Rotiload (Carl Roth). To detect γ-ENaC cleavage fragments, protein samples were deglycosylated with PNGase F according to the manufacturer’s instructions (New England Biolabs).

Subsequently, western blot analysis of α-, β-, and γ-ENaC was performed essentially as described previously [24, 63, 64]. Samples were boiled for 5 min at 95 °C and subjected to 10–12% SDS-PAGE. After separation, proteins were transferred to polyvinylidene difluoride membranes by semidry electroblotting and probed with subunit specific antibodies raised against an N-terminal epitope of α-ENaC (aa 20–42: LMKGNKREEQGLGPEPAAPQQPT), a C-terminal epitope of β-ENaC (aa 619–640: NYDSLRLQPLDVIESDSEGDAI), or a C-terminal epitope of γ-ENaC (aa 628–649: NTLRLERAFSNQLTDTQMLDEL) at a dilution of 1:5000 essentially as described previously [16, 24, 37]. Horseradish peroxidase-labelled secondary goat anti-rabbit antibodies (catalog no.: G21234; Invitrogen) were used as secondary antibodies in a dilution of 1:50,000. To validate the separation of cell surface from intracellular proteins in biotinylation experiments, blots were stripped and reprobed using a polyclonal anti-β-actin antiserum (Sigma-Aldrich) at a dilution of 1:5000.

### Manual whole-cell patch-clamp recordings in HEK293 cells

For manual patch-clamp recordings, ENaC-HEK293 cells were seeded onto poly-l-lysine coated coverslips and cultured for 1–2 days as described above. Conventional whole-cell patch-clamp recordings were performed essentially as described previously [40, 41, 53]. Patch pipettes were pulled from borosilicate glass capillaries and had a tip diameter of ~ 1–1.5 µm after fire polishing. Pipettes were filled with potassium gluconate solution (in mM: 140 K-Gluconate, 5 NaCl, 2 EGTA, 10 HEPES, 2 MgATP, pH 7.2 adjusted with Tris). Sodium chloride solution (in mM: 150 NaCl, 4 KCl, 2 CaCl_2_, 2 MgCl_2_, 10 HEPES, pH 7.4 adjusted with Tris) was used as an external solution. In this bath solution, the pipette resistance averaged ∼4 MΩ. Seal resistance was > 2 GΩ, and series resistance (R_s_) averaged ∼9 MΩ. Membrane capacitance (*C*_m_) and *R*_s_ were estimated using the automated capacitance compensation procedure of the EPC- 9 amplifier (HEKA). *C*_m_ ranged from 12 to 71 pF. Cells were voltage clamped at a holding potential of − 60 mV. Current signals were filtered at 125 Hz and sampled at a rate of 500 Hz. Experiments were performed at room temperature in a flow chamber continuously perfused with bath solution. Solutions were applied using a gravity-fed system (ALA BPS-8). Recordings were analyzed using the software Nest-o-Patch (https://sourceforge.net/projects/nestopatch/) developed by Dr. V. Nesterov (Institut für Zelluläre und Molekulare Physiologie, Friedrich-Alexander-Universität Erlangen-Nürnberg, Erlangen, Germany). The amiloride-sensitive current (Δ*I*_ami_) was determined by subtracting the whole-cell current measured in the absence of amiloride from that measured in the presence of amiloride (5 μM).

### Automated patch-clamp recordings in HEK293 cells

#### Cell harvesting

Typically, cells were detached using TrypLE Express at 37 °C in a cell incubator according to Nanion’s standard protocols [46]. Only in the experiments shown in Fig. 2b Na-Citrate solution was used instead (in mM: 135 KCl, 15 mM Na-Citrate). Subsequently, a cell suspension with a cell density of approximately 300,000–500,000 cells/ml was prepared in a divalent-free and nominally sodium-free solution (Table 1; NMDG^+^). The cell suspension was then placed in the SyncroPatch 384 “cell hotel” at 15 °C and was kept shaking at 200 rpm. No noticeable changes in cell quality were observed for up to 5 h following the preparation of the cell suspension. Therefore, multiple APC measurements could be performed using the same cell suspension.

**Table 1:** Composition of recording solutions for APC experiments (in mM)

	**KF110**	**NMDG**^**+**^ **140/ divalent-free**	**NMDG**^**+**^ **140/2 Ca**^**2+**^	**NMDG**^**+**^ **140/ 10 Ca**^**2+**^	**External standard**	**NMDG**^**+**^ **35/105 Na**^**+**^
	Internal	External	External	External	External	External
HEPES^1^	10	10	10	10	10	10
NaCl	10				140	105
NMDG^2^-Cl		140	140	140		35
KCl	10	4	4	4	4	4
CaCl_2_			2	10	2	2
MgCl_2_			1	1	1	1
K-Gluconate						
EGTA^3^	10					
Glucose		5	5	5	5	5
KF	110					
pH	7.2	7.4	7.4	7.4	7.4	7.4

^1^2-[4-(2-Hydroxyethyl)piperazin- 1-yl]ethanesulfonic acid

^2^N-methyl-D-glucamine

^3^Ethylene glycol-bis(2-aminoethylether)-N,N,N′,N′-tetraacetic acid

For experiments shown in Fig. 3b, a cell recovery protocol was established. After detachment with TrypLE Express, cells were centrifuged at 1000*g* for 5 min, and the resulting pellet was resuspended in 50 ml of the standard ENaC-HEK293 culture medium equilibrated with 5% CO_2_ in a falcon tube. The sealed falcon tube was kept for 4 h at 37 °C in an incubator on a falcon roller. For the SyncroPatch 384 measurements, the cell suspension was centrifuged again at 1000*g* for 5 min. The cell pellet was resuspended in NMDG-based solution (Table 1; NMDG^+^ 140/divalent-free), and the cell suspension was subsequently transferred into the cell hotel.

#### Automated whole-cell patch-clamp recordings

Whole-cell patch-clamp recordings were conducted on the SyncroPatch 384, a high-throughput patch-clamp instrument, according to Nanion’s standard procedures [46, 47]. All recordings were performed at room temperature (21 °C) using planar borosilicate glass consumables, the NPC-384 chips. Either one-hole or multi-hole (with 1 or 4 holes per well) NPC-384-chips were used for recordings, as indicated in corresponding figure legends. Data acquisition was performed using PatchControl 384 (Nanion Technologies) software. Recordings were obtained at a constant holding potential of − 60 mV without leak current subtraction.

The SyncroPatch 384 is a 384 well-based system where cells and solutions are automatically aspirated from different solution reservoirs located at predefined positions and added to the wells by a liquid handling robot (Biomek i5; Beckman Coulter) individually into each well [46]. At the beginning of experiments, the NPC-384 is loaded to the system, and all wells of a chip were filled with an external solution similar to that in the cell hotel supplemented with 2 mM Ca^2+^ and 1 mM Mg^2+^ (Table 1; NMDG^+^ 140/2 Ca^2+^). A standard KF110 (Nanion) solution was used as an internal solution (Table 1; KF 110). After transferring the cells from the cell hotel into each well, seal formation was facilitated by a brief and transient application of an extracellular solution containing 10 mM Ca^2+^ (Table 1; NMDG^+^ 140/10 Ca) followed by a washing step with NMDG^+^ 140/2 Ca^2+^ solution (Table 1). Thereafter, recordings were started in this sodium-free, NMDG^+^ 140/2 Ca^2+^ solution. Subsequently, half of the external solution was replaced by a standard extracellular solution containing 140 mM Na^+^ (Table 1; External Standard) yielding a nominal Na^+^ concentration of 70 mM in the well. This procedure was repeated a second time to achieve a final extracellular Na^+^ concentration of approximately 105 mM. Subsequently, half of the extracellular solution was replaced by NMDG^+^ 35/105 Na^+^ (Table 1) solution to ensure that a stable concentration of ~ 105 mM Na^+^ was reached in the bath solution as indicated in Figs. 2 and 3. All compounds were prepared and applied in NMDG^+^ 35/105 Na^+^ bath solution (Table 1).

#### Quality control parameters for automated whole-cell patch-clamp recordings

In order to ensure data quality and reproducibility, we documented quality control parameters using the recording software (PatchControl 384; Nanion Technologies). Individual recordings were only considered valid for the analysis when they met the following quality criteria:

(i) The initial seal resistance (in nominally sodium-free solution) and the final seal resistance at the end of the recording (in presence of amiloride) were > 50 MΩ for NPC-384 Chips with 4-holes per well or > 200 MΩ for NPC-384 Chips with 1-hole; similar quality criteria were used in previous studies with SyncroPatch384 [48, 52, 57].
(ii) The current recording was stable during the measurement.
(iii) The inhibitory effect of an ENaC inhibitor (amiloride and/or γ-−11 inhibitory peptide) was > 5 pA.

The success rate was calculated as the percentage of wells that met all the specified criteria out of all attempted recordings for each experimental condition.

### Two-electrode voltage-clamp experiments in *Xenopus laevis* oocytes

Full-length complementary DNAs (cDNAs) encoding human α-, β-, and γ-ENaC were originally obtained from Harry Cuppens [1]. cDNAs were subcloned into the pGEM-HE vector for heterologous expression in *X. laevis* oocytes. Plasmids were linearized using MluI restriction enzymes (MluI-HF, New England Biolabs) and used as templates for cRNA synthesis using SP6 RNA polymerase (mMessage mMachine, Ambion).

Isolation of oocytes was essentially performed as described previously [24, 62, 64, 65]. Ovarian lobes were excised by partial ovariectomy under anesthesia with Tricain 0.2%, in accordance with the principles of German legislation, with approval by the animal welfare officer for the University of Erlangen-Nürnberg (FAU), and under the governance of the state veterinary health inspectorate. Oocytes were isolated from ovarian lobes using a type-2 collagenase from *Clostridium histolyticum* (Sigma-Aldrich). Defolliculated stage V–VI oocytes were injected with 0.03 ng of cRNA per ENaC subunit (α, β, and γ) per oocyte. After cRNA injection, oocytes were kept in a low sodium ND9 solution (composition in millimolar: 9 NaCl, 2 KCl, 87 N-methyl-D-glutamine-Cl, 1.8 CaCl_2_, 1 MgCl_2_, 5 HEPES, and pH 7.4 adjusted with Tris) supplemented with 100 units/ml sodium penicillin and 100 μg/ml streptomycin sulfate. Two-electrode voltage-clamp measurements were performed 48 h after cRNA injection essentially as described previously [24, 62, 64, 65]. Bath solution exchanges with a gravity-fed system were controlled by a magnetic valve system (ALA BPS-8; ALA Scientific Instruments). Oocytes were clamped at a holding potential of − 60 mV using an OC-725 C amplifier (Warner Instruments) connected by an LIH-1600 (HEKA) to a personal computer. Pulse 8.78 (https://www.heka.com/) software (HEKA) was used for data acquisition. ND96 was used as a standard bath solution (composition in millimolar: 96 NaCl, 2 KCl, 1.8 CaCl_2_, 1 MgCl_2_, 5 Hepes; pH 7.4 adjusted with Tris). To test the effect of different cell detachment reagents, ENaC-mediated currents were assessed twice in each individual oocyte: before and after incubation of an oocyte with the respective reagent for 5 min directly in the superfusion chamber. During the incubation time, the membrane potential was not clamped to avoid Na^+^ overload.

### Statistical methods

Data are presented as mean ± SEM. Normal distribution of data was assessed using the D’Agostino-Pearson omnibus or Shapiro-Wilk test. Statistical significance was assessed using appropriate tests as indicated in figure legends. Automated patch-clamp recordings were analyzed using DataControl 384 (Nanion Technologies). Statistical analysis and figure preparation were performed using R environment for statistical computing v4.4.1 (R Core Team).

## Results

### Biochemical and functional characterization of ENaC stably expressed in a HEK293 cell line

To establish APC technique for electrophysiological recordings of ENaC, we obtained a commercially available HEK293 cell line with constitutive expression of human α-, β-, and γ-ENaC (ENaC-HEK293). To verify ENaC expression in these cells, we used biochemical methods and manual patch-clamp recordings.

**Fig. 1:**
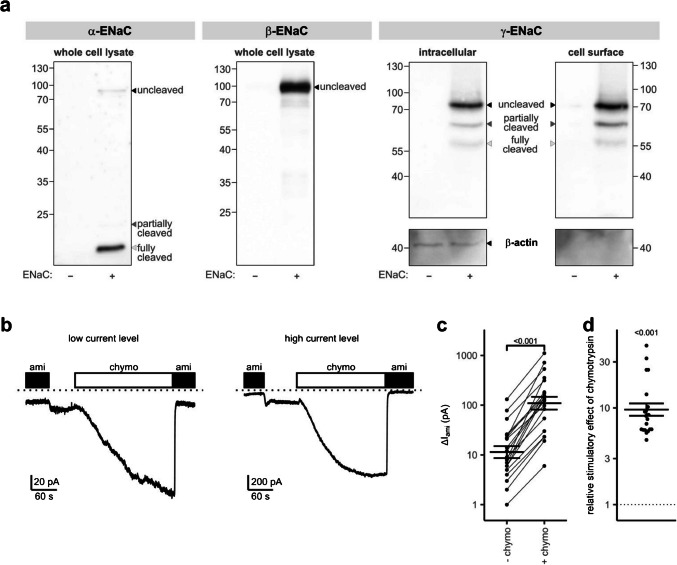
Characterization of ENaC expression and function in a HEK293 cell line. **a**–**c** Representative western blots showing expression of α- (*first panel*), β- (*second panel*), and γ-ENaC (*third and fourth panels*) in whole-cell lysates, intracellular or cell surface fractions as indicated. Commercially available ENaC-HEK293 cells stably transfected with human αβγ-ENaC (+) were used in parallel with control HEK293 cells without ENaC expression (−). Signals of uncleaved, partially cleaved and fully cleaved ENaC are marked with black, dark, and light gray arrowheads, respectively. Separation of cell surface from intracellular proteins in *third and fourth panels* was validated by stripping and re-probing the blots for β-actin. **b** Representative whole-cell manual patch-clamp recordings from ENaC-HEK293 cells with low (*left trace*) or high current levels (*right trace*). Amiloride (ami, 5 µM) and chymotrypsin (chymo, 5 µg/ml) were present in the bath solution as indicated by black and white bars, respectively. The dotted lines indicate zero current levels. **c** ENaC-mediated amiloride-sensitive whole-cell currents (Δ*I*_ami_) were determined from similar experiments as shown in **b** before (− chymo) or after application of chymotrypsin (+ chymo) by subtracting the current level reached in the absence of amiloride from the current level in the presence of amiloride. Lines connect datapoints obtained in the same recording. Mean ± SEM and individual datapoints are shown on a logarithmic scale; two-sided paired Student’s *t*-test (*n* = 20). **d** Relative stimulatory effect of chymotrypsin on Δ*I*_ami_ calculated from data shown in **c**. The dotted line indicates a relative effect of one (no effect). Mean ± SEM and individual datapoints are shown on a logarithmic scale; one-sample, two-sided Student’s *t*-test of logarithmic values

Immunoblotting experiments confirmed protein expression of all three ENaC subunits in this cell line (Fig. 1a) and also allowed the assessment of proteolytic cleavage of the different subunits. Using an antibody targeting an N-terminal epitope of α-ENaC, we observed a strong 15 kDa band, which was absent in control HEK293 cells without ENaC expression. This band most likely represents an α-ENaC fragment resulting from intracellular processing of ENaC by furin or furin-like convertases [16, 22, 23, 63]. We also detected two additional faint signals at 80 kDa and 20 kDa, which probably correspond to a full-length and a partially cleaved form of α-ENaC, respectively. Thus, α-ENaC was present in ENaC-HEK293 cells mainly in its fully cleaved form. For β-ENaC, which is not subject to proteolytic cleavage, a single band at 100 kDa was observed using an antibody directed against a C-terminal sequence. Finally, we used an antibody targeting a C-terminal epitope of γ-ENaC that was previously used successfully to detect full length, partially cleaved, and fully cleaved forms of γ-ENaC [63, 64]. Importantly, in both intracellular and cell surface fractions of ENaC-HEK293 cells, mainly full-length γ-ENaC at 70 kDa was detected, whereas the signals corresponding to partially cleaved (60 kDa) and fully cleaved (55 kDa) γ-ENaC were considerably weaker. In summary, these experiments confirmed the expression of α-, β-, and γ-ENaC in ENaC-HEK293 cells. Whereas α-ENaC was present mainly in its fully cleaved form, the γ-subunit appeared to be predominantly expressed in its uncleaved, full-length form.

Next, ENaC was functionally assessed in these cells using conventional manual whole-cell patch-clamp technique. Recordings started in the presence of the ENaC inhibitor amiloride (5 µM; Fig. 1b) that is known to block ENaC-mediated currents almost completely at this concentration [34]. Washout of amiloride revealed small Na^+^ inward currents averaging 23 ± 7 pA (Fig. 1b, c). Notably, the magnitude of these currents varied significantly in a range between 1 and 131 pA, probably reflecting cell-to-cell variability in ENaC expression. Regardless of the level of baseline ENaC currents, extracellular application of the prototypical protease chymotrypsin (5 µg/ml) resulted in a robust stimulation of the inward currents by about tenfold (Fig. 1b–d). This probably represents the conversion of uncleaved and/or partially cleaved γ-ENaC into the fully cleaved form. Subsequent application of amiloride returned currents to their initial levels, confirming that the inward current increase in the presence of chymotrypsin was due to proteolytic ENaC activation. Importantly, despite variable ENaC baseline currents, the qualitative current response of ENaC-HEK293 cells to chymotrypsin was highly reproducible.

We therefore conclude that ENaC-HEK293 cells express functional human αβγ-ENaC and are potentially suitable for APC measurements. Interestingly, γ-ENaC was found to be predominantly uncleaved in these cells which likely explains the relatively low baseline amiloride-sensitive currents and the large stimulatory effect of chymotrypsin in our manual patch-clamp experiments.

### Enzymatic cell detachment allows high-throughput APC recordings of ENaC and leads to partial proteolytic ENaC activation

Next, we wanted to establish a protocol for high-throughput APC recordings of ENaC using the SyncroPatch 384. For these experiments, cultured ENaC-HEK293 cells were detached into a single cell suspension with the use of the proteolytic enzyme TrypLE (a recombinant trypsin-like protease). This approach is called “harvesting” and is commonly used in APC for detachment of a wide range of cells including HEK293. A possible side-effect of this cell harvesting protocol may be proteolytic ENaC activation. To test the ability of TrypLE to cause proteolytic ENaC activation, we performed two-electrode voltage-clamp recordings of human αβγENaC heterologously expressed in *Xenopus laevis* oocytes. Application of TrypLE for 5 min caused complete proteolytic activation of the channel (Supplemental Fig. S1) as evidenced by significantly increased amiloride-sensitive currents (Δ*I*_ami_) and the absence of an additional stimulatory effect of chymotrypsin after TrypLE treatment. Notably, the stimulatory effect of TrypLE on ENaC was similar to that of other enzymatic cell detachment reagents like trypsin, collagenase, and dispase (Supplemental Fig. S1). In contrast, incubation of oocytes in sodium citrate or EDTA solution, which can be used for enzyme-free cell harvesting, did not affect ENaC-mediated currents with subsequent chymotrypsin application producing a typical ~ twofold stimulation of Δ*I*_ami_ (Supplemental Fig. S1). This indicated that, as expected, these non-enzymatic cell detachment solutions did not cause proteolytic ENaC activation.

**Fig. 2:**
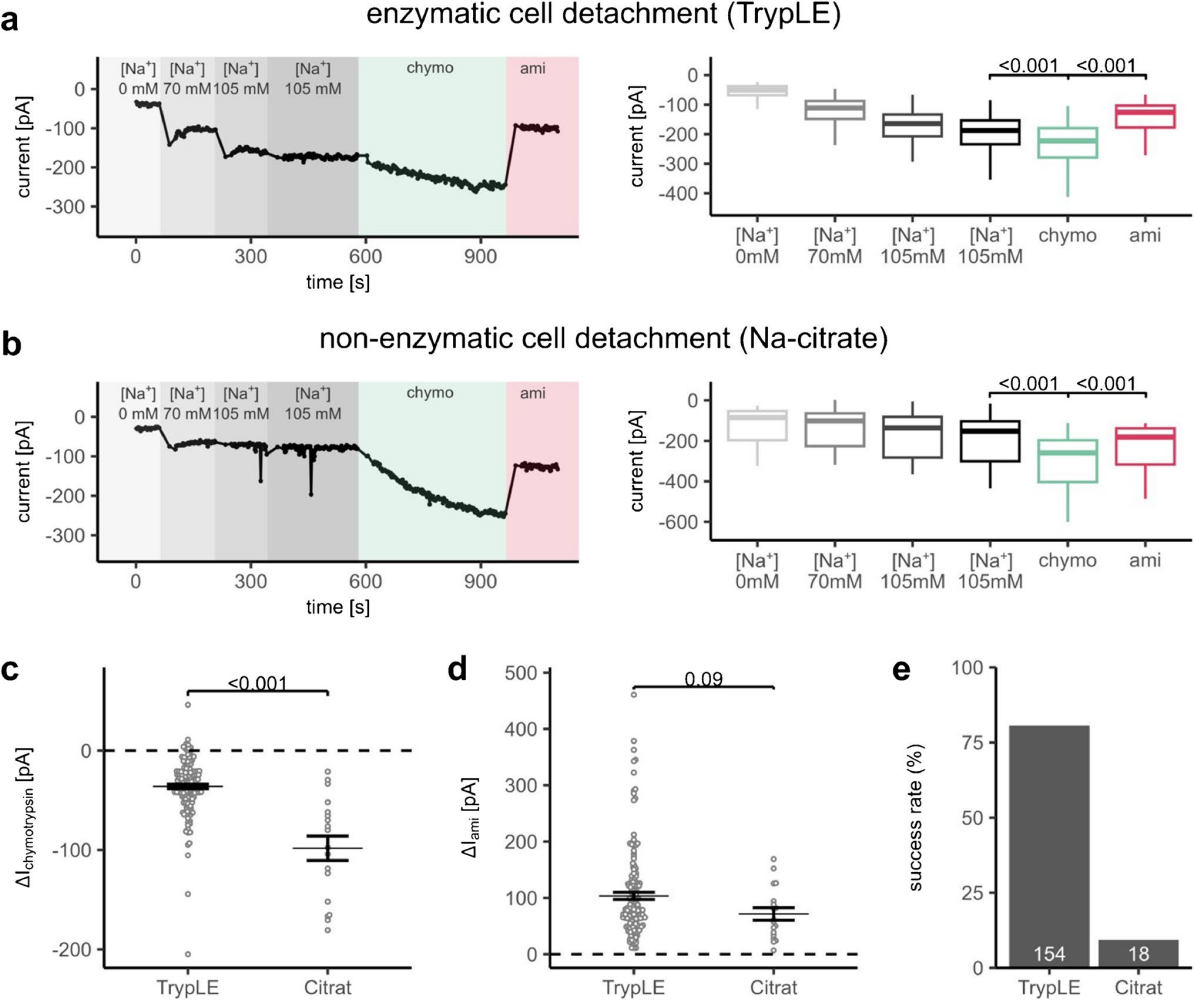
APC recordings from human ENaC expressing HEK293 cells after enzymatic or non-enzymatic cell detachment. **a**, **b** *Left panels*: representative APC recordings after cell detachment with TrypLE (**a**) or sodium citrate (**b**) obtained with a multi-hole chip (4 holes per well, 4 × S-Type; Nanion, product no.: 22 2401). Background colors and labels indicate different bath solutions. Measurements were started in nominally Na^+^-free solution ([Na^+^] 0 mM), followed by solution exchanges to increase [Na^+^] to 70 mM ([Na^+^] 70 mM) and then to 105 mM ([Na^+^] 105 mM) as explained in methods. An additional solution exchange step was performed with an extracellular solution containing 105 mM Na^+^ to confirm that a current plateau was reached. Green and violet indicate subsequent applications of chymotrypsin (10 µg/ml, chymo) and amiloride (10 µM, ami), respectively. *Right panels:* summary data from similar experiments as shown in the corresponding *left panels*. Current levels were determined in each individual trace at the end of the respective solution application phase by averaging the final 5 data points. Box-Whisker-Plots indicate median (bold horizontal line), first and third quartile (box), minimum and maximum values (whiskers) excluding outliers greater than 1.5 interquartile ranges outside the first and third quartile. Two-sided, paired Student’s *t*-test (**a**: *n* = 154, **b**: *n* = 18). **c**, **d** Absolute effect of chymotrypsin (**c**, Δ*I*_chymotrypsin_) or subsequently applied amiloride (**d**, Δ*I*_ami_) on currents calculated from data shown in (**a**, **b**). Values were obtained by subtracting the current level reached before chymotrypsin (**c**) or amiloride (**d**) application from the value reached in the presence of chymotrypsin (**c**) or amiloride (**d**). The dotted line indicates an absolute effect of zero (no effect). Mean ± SEM and individual datapoints are shown. Two-sided Student’s *t*-test. **e** The ratio of successful recordings to all recordings in the respective experimental group. A recording was considered successful when (i) the seal resistance was > 50 MΩ, (ii) the current recording was stable during the measurement, and (iii) the inhibitory effect of amiloride was > 5 pA. Numbers in white within each bar represent the number of successful recordings per group

We compared enzymatic (TrypLE-based) and non-enzymatic (sodium citrate-based) cell detachment protocols regarding their suitability for APC measurements and their effect on proteolytic ENaC activation in ENaC-HEK293 cells. Figure 2a shows a representative whole-cell APC current recording obtained in a cell harvested with TrypLE at a continuous holding potential of − 60 mV. Measurements were started in a nominally Na^+^ free extracellular solution ([Na^+^] 0 mM) to prevent Na^+^ inward currents and overloading of cells with Na^+^. This solution was prepared on the basis of a standard extracellular solution (see Table 1) by substituting 140 mM of Na^+^ equimolarly by a large organic cation NMDG^+^ for which ENaC is impermeable. Subsequently, roughly half of the extracellular solution was exchanged with a 140 mM Na^+^ solution, resulting in ~ 70 mM extracellular Na^+^ concentration. A second partial solution exchange with 140 mM Na^+^ solution increased extracellular Na^+^ further to ~ 105 mM. This Na^+^ concentration is sufficient to achieve nearly maximal ENaC-mediated inward currents in whole-cell recordings [33]. As expected, this stepwise increase in the extracellular Na^+^ concentration was paralleled by a stepwise increase in the measured inward currents, suggesting expression of active ENaC in the cell plasma membrane. An additional subsequent solution exchange with a solution containing 105 mM Na^+^ was performed to ensure that the Na^+^ concentration in the bath had reached a plateau at ~ 105 mM. Indeed, this maneuver did not substantially alter the magnitude of Na^+^ inward currents (Fig. 2a, b). To test whether TrypLE incubation resulted in full or only partial proteolytic ENaC activation, chymotrypsin (10 µg/ml) was applied. Chymotrypsin application caused a further increase of inward currents (Fig. 2a, c). However, the relative stimulatory effect of chymotrypsin in APC measurements from cells detached with TrypLE was considerably lower than that observed in experiments using manual patch-clamp (Fig. 1b–d). These findings suggested that cell detachment with TrypLE caused partial proteolytic ENaC activation. Application of amiloride (10 µM) at the end of the recording substantially inhibited inward Na^+^ currents. At this concentration, amiloride is reported to inhibit ENaC-mediated currents by more than 95% [27, 34]. Therefore, the remaining current in the presence of amiloride can be attributed to background or leak currents, which are not ENaC-specific. Nevertheless, the pronounced inhibitory effect of amiloride confirmed that the observed inward currents were at least in part mediated by ENaC and that the stimulatory effect of chymotrypsin was due to additional proteolytic ENaC stimulation.

In cells harvested using the non-enzymatic cell detachment protocol, the baseline Na^+^ inward currents appeared to be lower and showed a more pronounced stimulatory response to chymotrypsin (Fig. 2b, c). Indeed, the current increase in the presence of chymotrypsin in cells detached with citrate averaged − 98.2 ± 2.9 pA, which was more than 2.5-fold higher than in cells detached with TrypLE (− 36.1 ± 0.2 pA; Fig. 2c). The inhibitory effect of amiloride was not significantly different between both groups (103.5 ± 0.5 pA *vs.* 71.5 ± 2.6 pA in TrypLE and citrate group, respectively; Fig. 2d). Thus, ENaC expression and its activity after chymotrypsin treatment were similar in both experimental groups.

It should be noted that only recordings which fulfilled certain quality criteria were included in the summary data shown in Fig. 2. As highlighted in the methods section, a recording was considered successful when (i) the seal resistance was > 50 MΩ, (ii) the current recording was stable during the measurement, and (iii) the inhibitory effect of amiloride was > 5 pA. The latter was to ensure that ENaC expression was sufficient to resolve ENaC-mediated currents. Importantly, ~ 75% of all recordings in cells detached with TrypLE matched these criteria, whereas the success rate was < 10% in citrate-treated cells (Fig. 2e).

Taken together, these results indicate that the non-enzymatic cell detachment with citrate results in lower success rate of APC recordings but offers the opportunity of detecting large stimulatory effects of channel activating proteases. In contrast, the high success rate with enzymatic cell detachment with TrypLE makes this approach more suitable for high-throughput APC recordings. Importantly, the partial proteolytic ENaC activation due to the use of TrypLE for cell detachment did not prevent detection of additional proteolytic ENaC activation by chymotrypsin. Nevertheless, the observed partial proteolytic pre-activation of ENaC may reduce the sensitivity of APC recordings with TrypLE detached cells to identify ENaC activators mimicking proteolytic channel activation.

### An improved protocol for better resolution of proteolytic ENaC activation in APC recordings

We hypothesized that keeping cells suspended in culture medium at 37 °C for several hours after enzymatic cell detachment with TrypLE may improve the detection of proteolytic ENaC activation in APC measurements. We expected that during this recovery time, cells will internalize proteolytically cleaved ENaC while building up a pool of newly inserted uncleaved ENaC at the cell surface.

**Fig. 3:**
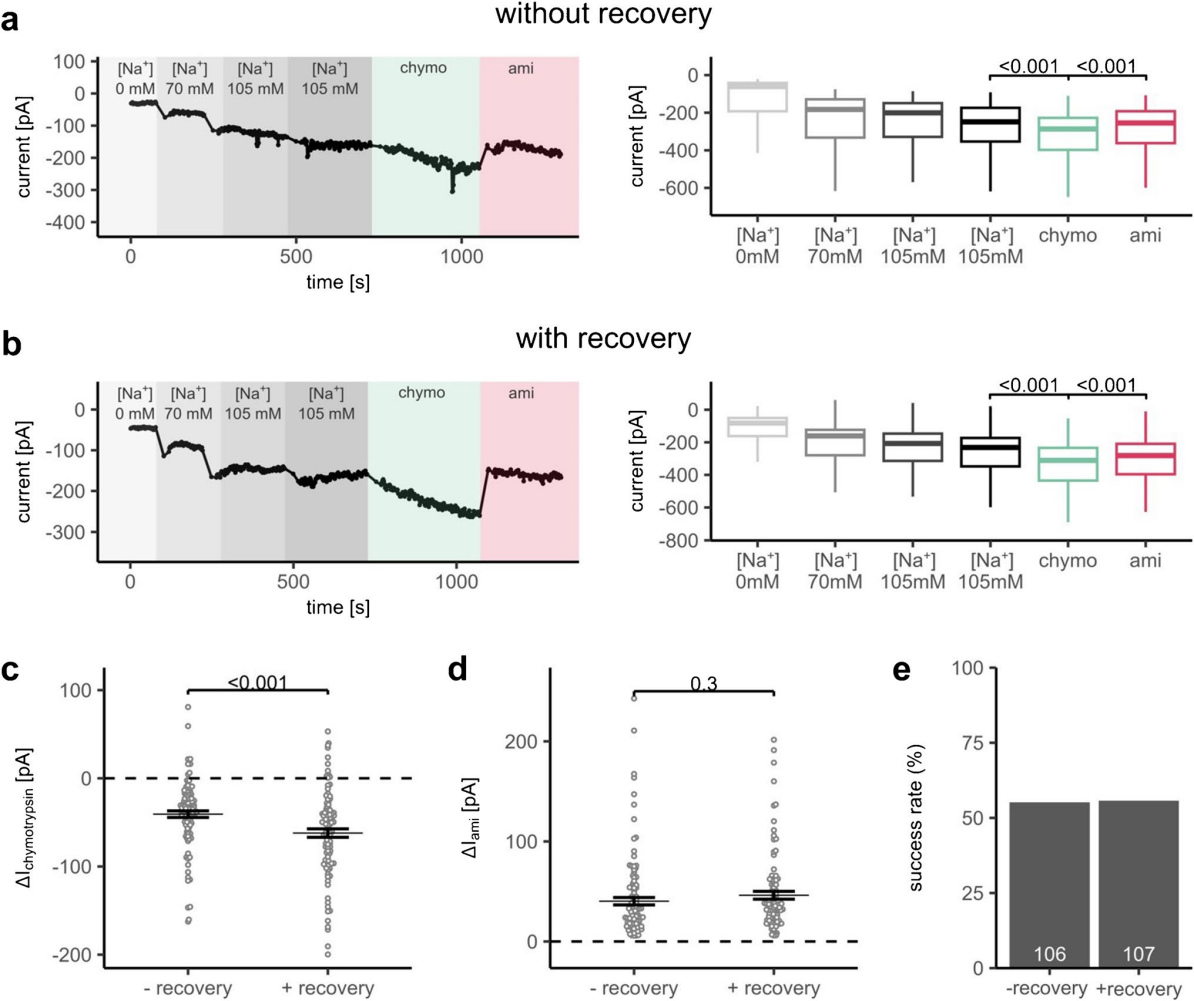
A recovery period after enzymatic cell detachment enhances the relative stimulatory effect of chymotrypsin on ENaC currents in APC recordings. **a**, **b** *Left panels*: representative APC recordings obtained directly after cell detachment with TrypLE (**a**; without recovery) or after 4 h of recovery in complete medium at 37 °C (**b**; with recovery) using a multi-hole chip (4 holes per well, 4 × S-Type; Nanion, product no.: 22 2401). Background colors and labels indicate bath solutions as described in Fig. 2a, b. *Right panels*: summary data from similar experiments as in the corresponding *left panels*. Current levels were determined as described in Fig. 2a, b. Two-sided, paired Student’s *t*-test (**a**: *n* = 106, **b**: *n* = 107). **c**, **d** Absolute effect of chymotrypsin (**c**, Δ*I*_chymotrypsin_) or subsequently applied amiloride (**d**, Δ*I*_ami_) on currents measured in cells without (−) or with (+) recovery step was calculated from data shown in **a**, **b** as described in Fig. 2c, d. The dotted line indicates an absolute effect of zero (no effect). Mean ± SEM and individual data points are shown. Two-sided Student’s *t*-test. **e** The ratio of successful recordings to all recordings in the respective experimental group, calculated as described in Fig. 2e. Numbers in white within each bar represent the number of successful recordings per group

Consistent with this hypothesis, the stimulatory effect of chymotrypsin on cells after 4 h of recovery in culture medium was significantly increased (− 62.1 ± 0.5 pA; Fig. 3b, c) compared to that in freshly detached cells (− 39.9 ± 0.4 pA; Fig. 3a, c). Thus, the recovery protocol improved the detection of proteolytic ENaC activation. The effect of subsequently applied amiloride was not significantly different between the two groups, as expected (44.3 ± 0.4 and 41.7 ± 0.4 pA with or without recovery, respectively; Fig. 3d). Importantly, unlike the non-enzymatic cell detachment protocol (Fig. 2e), the recovery protocol did not have a negative impact on the success rate of the measurements (Fig. 3e).

To conclude, this simple recovery protocol may be useful to facilitate the identification of novel ENaC activators stimulating the channel via molecular mechanisms similar to those of proteases.

### High-throughput detection of ligand-based ENaC modulation using the APC technique

**Fig. 4:**
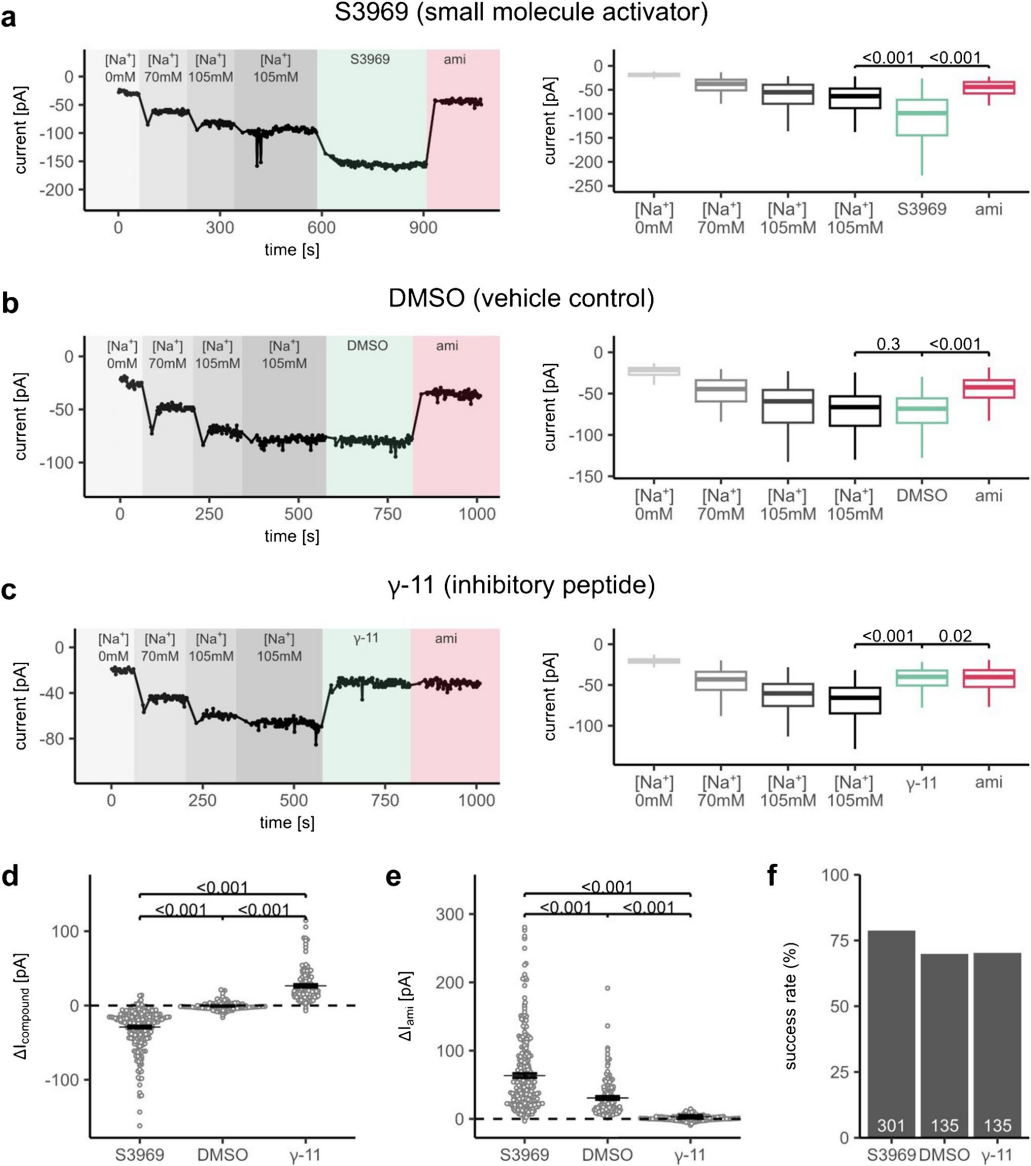
APC recordings are suitable to detect stimulatory and inhibitory effects of known ENaC modulators on ENaC currents. **a**–**c** *Left panels:* representative APC recordings obtained with a single-hole chip (1 hole per well, 1 × S-Type; Nanion, product no.: 22 2101) demonstrating effects of the small-molecule ENaC-activator S3969 (**a**), vehicle-control DMSO (**b**) and the synthetic ENaC-inhibiting 11-mer peptide, γ-11 (**c**). Background colors and labels indicate different bath solutions. Measurements started with a similar solution exchange protocol as described in Fig. 2a, b. Green indicates subsequent application of S3969 (**a**; S3969, 10 µM), the vehicle-control DMSO (**b**; DMSO, 0.01% v/v), or the inhibitory peptide (**c**; γ-11, 10 µM). Violet indicates application of amiloride at the end of the recording (10 µM, ami). *Right panels*: summary data from similar experiments as in the corresponding *left panels*. Current levels were determined at the end of each phase, as described in Fig. 2a, b. Two-sided, paired Student’s *t*-test (**a**: *n* = 301, **b**: *n* = 135, **c**: *n* = 135). **d**, **e** Absolute effect of S3969, DMSO, or γ-11 (**d**, Δ*I*_compound_) or subsequently applied amiloride (**d**, Δ*I*_ami_) on currents calculated from data shown in **a**, **b** similarly as described in Fig. 2c, d. The dotted line indicates an absolute effect of zero (no effect). Mean ± SEM and individual datapoints are shown. Two-sided Student’s *t*-test. **f** The ratio of successful recordings to all recordings in the respective experimental group, calculated as described in Fig. 2e with the following modification in criterium no. (iii): the inhibitory effect of either the applied compound or amiloride was > 5 pA. Numbers in white within each bar represent the absolute number of successful recordings per group. Note that the total number of recordings was different between groups

Finally, we wanted to confirm the suitability of our approach for identifying both positive and negative modulators of ENaC in APC recordings. In these proof-of-principle experiments, we used a well-established small molecule ENaC activator S3969 to stimulate ENaC. The γ-ENaC inhibitory peptide (γ-11) or amiloride were used as established ENaC inhibitors. Cells were studied directly after harvesting with TrypLE without an additional recovery step. Application of the ENaC-activator S3969 (10 µM) resulted in a prompt increase of the inward currents by − 28.9 ± 0.1 pA (Fig. 4a, d). This stimulatory effect was fully blocked by application of amiloride at the end of the recording, confirming that it was due to ENaC activation. In parallel control recordings, we demonstrated that application of a vehicle (DMSO, 0.01% v/v) did not significantly change ENaC-mediated currents (Fig. 4b, d). Finally, we reliably detected an inhibitory effect of γ-11 (10) on ENaC currents (Fig. 4c, d). Application of γ-11 resulted in a significant reduction of the inward currents, on average by 26.6 ± 0.2 pA (Fig. 4d). Indeed, in the presence of γ-11, ENaC-mediated currents were essentially abolished, as evidenced by the absence of an additional inhibitory effect of amiloride applied in the presence of γ-11 (Fig. 4c, e). Consistent with our other APC experiments, the probability of obtaining a successful ENaC recording was > 70% in these experiments (Fig. 4f). Essentially identical results were obtained in an additional set of experiments using a different cell preparation (Fig. S2).

Taken together, these proof-of-principle experiments demonstrated that APC recordings with the SyncroPatch 384 system are suitable for the identification of ENaC activators and inhibitors in human ENaC expressing HEK293 cells.

## Discussion

In this study, we made the following key observations: (1) commercially available ENaC-HEK293 cells expressed functional human αβγ-ENaC; (2) in these cells, only α-ENaC was fully cleaved by endogenous proteases, while γ-ENaC was predominantly found in its uncleaved form; this latter finding probably explains the large (~ tenfold) stimulatory effect of chymotrypsin on ENaC whole-cell currents observed in manual patch-clamp recordings; (3) a TrypLE-based protocol for enzymatic cell detachment led to partial proteolytic activation of ENaC, reducing the stimulatory effect of exogenous chymotrypsin in APC recordings; (4) proof-of-principle experiments demonstrated that allowing cells to recover after TrypLE treatment improved the detection of proteolytic ENaC activation; (5) even without using this cell recovery protocol, APC recordings with ENaC-HEK293 cells showed high success rates and reliably captured the effects of known ENaC modulators. We conclude that the experimental protocols established in this study provide a suitable basis for future high-throughput screening experiments to discover novel ENaC activators and inhibitors using the APC technique.

A cell line with stable functional expression of ENaC is a fundamental prerequisite for successful APC measurements of ENaC-mediated currents. Our western blot analysis confirmed that ENaC-HEK293 cells expressed α-, β-, and γ-ENaC subunits. According to the current paradigm, intracellular maturation of ENaC involves two proteolytic cleavage events in α-ENaC and one in γ-ENaC [22, 23]. The second cleavage event in γ-ENaC appears to be critical for full proteolytic channel activation and probably occurs at the cell surface [8, 15, 29, 30]. While α-ENaC was predominantly detected in its fully cleaved form in ENaC-HEK293 cells, we found γ-ENaC mostly in its uncleaved state. Consistent with these findings, we observed relatively low baseline ENaC currents that could be increased by ~ tenfold upon chymotrypsin application in manual patch-clamp recordings of these cells. Similar observations were made previously by Ruffieux-Daidié et al., who observed low ENaC currents and detected predominant cleavage of α- but not γ-ENaC in a HEK293 cell line stably transfected with αβγ-ENaC [59]. Intracellular cleavage of α- and γ-ENaC is believed to be performed by furin or related convertases [22, 23]. The predominant cleavage of α- but not γ-ENaC may reflect the presence of distinct proteases responsible for processing these two ENaC subunits. It is conceivable that in ENaC-HEK293 cells, the expression of proteases required for complete cleavage of γ-ENaC is insufficient. Alternatively, overexpression of ENaC could saturate trafficking or maturation pathways, allowing for complete α- but not γ-ENaC processing. Furthermore, Ruffieux-Daidié et al. proposed that ubiquitination and subsequent internalization of γ-ENaC may interfere with proteolytic cleavage of this subunit in a HEK293 cell model. Indeed, coexpression of the deubiquitylation enzyme Usp2-45 was shown to enhance γ-ENaC processing and increase ENaC-mediated currents [59]. Regardless of these peculiarities in proteolytic ENaC processing by HEK293 cells, we can conclude that commercially available ENaC-HEK293 cells functionally express human ENaC and are suitable for APC measurements.

For APC measurements, adherent cells are converted into a single cell suspension [46]. Typically, this is achieved by incubating the cells with enzymatic cell detachment reagents that degrade extracellular matrix, tight-junctions, and other cell adhesion proteins [13, 54]. This treatment can also result in proteolytic digestion of cell surface proteins including ion channels or other transmembrane receptors, as demonstrated previously [20, 28, 31, 51]. In our study, we observed that a commonly used enzymatic cell detachment reagent, TrypLE, acutely caused full proteolytic ENaC activation in the oocyte expression system and partial proteolytic ENaC activation in cultured cells detached with TrypLE. This significantly reduced the relative stimulatory effect of chymotrypsin in APC recordings compared to manual patch-clamp experiments carried out with adherent cells and thus without usage of TrypLE. To improve the detection of proteolytic ENaC activation in APC experiments, we explored two methodological approaches. First, we tested alternative detachment protocols. However, experiments in *X. laevis* oocytes indicated that other commonly used enzymatic cell detachment reagents, like trypsin, collagenase, and dispase [4], also proteolytically activate ENaC similar to TrypLE. In contrast, non-enzymatic detachment solutions, e.g., sodium citrate or EDTA, which chelate Ca^2+^ to disrupt cadherin-mediated cell–cell adhesion [66, 66], did not proteolytically activate ENaC in oocytes or in HEK293 cells. Citrate-based cell detachment was also applied for APC measurements and resulted in a higher stimulatory effect of chymotrypsin similar to that seen in our manual patch-clamp recordings. However, citrate-based cell detachment resulted in a rather low success rate in APC measurements, making this approach less suitable for high-throughput screening. To obtain a single-cell suspension using sodium citrate, application of additional mechanical forces was necessary (i.e., repeated pipetting to break up cell clusters). This maneuver likely caused cell damage but was inefficient for proper singularization of cells, which might explain the low success rate of APC recordings in cells detached with citrate. We hypothesized that keeping cells in suspension after TrypLE treatment for several hours at 37 °C in culture medium would result in internalization of cleaved ENaC and replacement by newly inserted uncleaved ENaC. A similar strategy was implemented previously for cell recovery after enzymatic treatment [31]. Consistent with our hypothesis, the cell recovery approach significantly increased the stimulatory effect of chymotrypsin in proof-of-principle APC experiments. Thus, this approach can be considered as an option when searching specifically for modulators mimicking proteolytic ENaC activation.

Importantly, the standard TrypLE-based detachment protocol, even without the recovery step, enabled reliable detection of well-established ENaC modulators in APC experiments and yielded very high success rates. Notably, baseline ENaC currents in ENaC-HEK293 cells were only about 20 pA, which may hamper the detection of ENaC inhibitors. Proteolytic stimulation of ENaC by TrypLE increased baseline currents, thereby improving the resolution of the inhibitory effects of amiloride and γ-11 peptide on ENaC. Thus, cell detachment with TrypLE may even be beneficial for identifying novel ENaC inhibitors. Interestingly, the stimulatory effect of S3969 could also be successfully detected even after partial proteolytic activation of the channel by TrypLE. This suggests that S3969 activates ENaC via a molecular mechanism distinct from that triggered by proteolytic cleavage, which is consistent with our previous observations [65].

In future studies, it would be interesting to assess the applicability of the APC technique in native epithelial cells endogenously expressing ENaC, such as principal cortical collecting duct or lung epithelial cells. Notably, APC recordings have been successfully conducted in several primary and stem cell-derived cell lines, including human-induced pluripotent stem cell-derived cardiomyocytes (hiPSC-CMs) [48]. Ideally, the cell line should be of human origin to enable the use of S3969 as an ENaC activator, because this compound stimulates human but not rodent ENaC [34, 65]. The latter finding highlights the relevance of species differences when screening for novel ENaC modulators.

In summary, we have demonstrated the feasibility of using APC recordings with the SyncroPatch 384 system to detect reliably the effects of activators and/or inhibitors of ENaC currents in HEK293 cells expressing human ENaC. Our study offers a methodological basis for future screening experiments to identify novel endogenous or pharmacological ENaC activators and inhibitors. The discovery of novel ENaC modulators eventually may lead to new therapeutic options to treat diseases associated with pathologically altered ENaC activity, including hypertension, cystic fibrosis, pulmonary edema and respiratory distress.

## Supplementary Information

Below is the link to the electronic supplementary material.

Supplementary file1 (DOCX 2141 KB)

Supplementary file2 (DOCX 993 KB)

## Data Availability

No datasets were generated or analysed during the current study.

## References

[1] Azad AK, Rauh R, Vermeulen F, Jaspers M, Korbmacher J, Boissier B, Bassinet L, Fichou Y, Georges Des M, Stanke F, De Boeck K, Dupont L, Balascakova M, Hjelte L, Lebecque P, Radojkovic D, Castellani C, Schwartz M, Stuhrmann M, Schwarz M, Skalicka V, de Monestrol I, Girodon E, Ferec C, Claustres M, Tummler B, Cassiman JJ, Korbmacher C, Cuppens H (2009) Mutations in the amiloride-sensitive epithelial sodium channel in patients with cystic fibrosis-like disease. Hum Mutat 30:1093–1103. 10.1002/humu.21011 19462466 10.1002/humu.21011

[2] Bohnert BN, Daiminger S, Worn M, Sure F, Staudner T, Ilyaskin AV, Batbouta F, Janessa A, Schneider JC, Essigke D, Kanse S, Haerteis S, Korbmacher C, Artunc F (2019) Urokinase-type plasminogen activator (uPA) is not essential for epithelial sodium channel (ENaC)-mediated sodium retention in experimental nephrotic syndrome. Acta Physiol (Oxf) 227:e13286. 10.1111/apha.13286 31006168 10.1111/apha.13286

[3] Boucher RC (2007) Airway surface dehydration in cystic fibrosis: pathogenesis and therapy. Annu Rev Med 58:157–170. 10.1146/annurev.med.58.071905.105316 17217330 10.1146/annurev.med.58.071905.105316

[4] Capes-Davis A, Capes-Davis A, Freshney RI (2021) Freshney’s culture of animal cells: a manual of basic technique and specialized applications. Eighth edition. edn, Wiley-Blackwell, Hoboken, NJ

[5] Chang SS, Grunder S, Hanukoglu A, Rosler A, Mathew PM, Hanukoglu I, Schild L, Lu Y, Shimkets RA, Nelson-Williams C, Rossier BC, Lifton RP (1996) Mutations in subunits of the epithelial sodium channel cause salt wasting with hyperkalaemic acidosis, pseudohypoaldosteronism type 1. Nat Genet 12:248–253. 10.1038/ng0396-248 8589714 10.1038/ng0396-248

[6] Danahay H, Gosling M, Fox R, Lilley S, Charlton H, Hargrave JD, Schofield TB, Hay DA, Went N, McMahon P, Marlin F, Scott J, Vile J, Hewison S, Ellam S, Brown S, Sabater J, Kennet G, Lightowler S, Collingwood SP (2025) Optimisation of a novel series of ENaC inhibitors, leading to the selection of the long-acting inhaled clinical candidate ETD001, a potential new treatment for cystic fibrosis. Eur J Med Chem 282:117040. 10.1016/j.ejmech.2024.117040 39561495 10.1016/j.ejmech.2024.117040

[7] Danahay H, McCarthy C, Schofield T, Fox R, Charlton H, Lilley S, Sabater J, Salathe M, Baumlin N, Collingwood SP, Gosling M (2024) ETD001: a novel inhaled ENaC blocker with an extended duration of action in vivo. J Cyst Fibros. 10.1016/j.jcf.2024.06.002 38851923 10.1016/j.jcf.2024.06.002

[8] Diakov A, Bera K, Mokrushina M, Krueger B, Korbmacher C (2008) Cleavage in the gamma-subunit of the epithelial sodium channel (ENaC) plays an important role in the proteolytic activation of near-silent channels. J Physiol 586:4587–4608. 10.1113/jphysiol.2008.154435 18669538 10.1113/jphysiol.2008.154435 PMC2614049

[9] Diakov A, Korbmacher C (2004) A novel pathway of epithelial sodium channel activation involves a serum- and glucocorticoid-inducible kinase consensus motif in the C terminus of the channel’s alpha-subunit. J Biol Chem 279:38134–38142. 10.1074/jbc.M403260200 15234985 10.1074/jbc.M403260200

[10] Diakov A, Nesterov V, Dahlmann A, Korbmacher C (2022) Two adjacent phosphorylation sites in the C-terminus of the channel’s alpha-subunit have opposing effects on epithelial sodium channel (ENaC) activity. Pflugers Arch 474:681–697. 10.1007/s00424-022-02693-9 35525869 10.1007/s00424-022-02693-9 PMC9192390

[11] Eaton DC, Helms MN, Koval M, Bao HF, Jain L (2009) The contribution of epithelial sodium channels to alveolar function in health and disease. Annu Rev Physiol 71:403–423. 10.1146/annurev.physiol.010908.163250 18831683 10.1146/annurev.physiol.010908.163250

[12] Fronius M (2013) Treatment of pulmonary edema by ENaC activators/stimulators. Curr Mol Pharmacol 6:13–27. 10.2174/1874467211306010003 23547931 10.2174/1874467211306010003

[13] Glazer ES, Massey KL, Curley SA (2010) A protocol to effectively create single cell suspensions of adherent cells for multiparameter high-throughput flow cytometry. In Vitro Cell Dev Biol Anim 46:97–101. 10.1007/s11626-009-9256-8 19997869 10.1007/s11626-009-9256-8

[14] Grunder S, Firsov D, Chang SS, Jaeger NF, Gautschi I, Schild L, Lifton RP, Rossier BC (1997) A mutation causing pseudohypoaldosteronism type 1 identifies a conserved glycine that is involved in the gating of the epithelial sodium channel. EMBO J 16:899–907. 10.1093/emboj/16.5.899 9118951 10.1093/emboj/16.5.899 PMC1169690

[15] Haerteis S, Krappitz M, Diakov A, Krappitz A, Rauh R, Korbmacher C (2012) Plasmin and chymotrypsin have distinct preferences for channel activating cleavage sites in the gamma subunit of the human epithelial sodium channel. J Gen Physiol 140:375–389. 10.1085/jgp.201110763 22966015 10.1085/jgp.201110763 PMC3457690

[16] Haerteis S, Krueger B, Korbmacher C, Rauh R (2009) The delta-subunit of the epithelial sodium channel (ENaC) enhances channel activity and alters proteolytic ENaC activation. J Biol Chem 284:29024–29040. 10.1074/jbc.M109.018945 19717556 10.1074/jbc.M109.018945 PMC2781449

[17] Hansson JH, Schild L, Lu Y, Wilson TA, Gautschi I, Shimkets R, Nelson-Williams C, Rossier BC, Lifton RP (1995) A de novo missense mutation of the beta subunit of the epithelial sodium channel causes hypertension and Liddle syndrome, identifying a proline-rich segment critical for regulation of channel activity. Proc Natl Acad Sci U S A 92:11495–11499. 10.1073/pnas.92.25.11495 8524790 10.1073/pnas.92.25.11495 PMC40428

[18] Hartmann EK, Boehme S, Duenges B, Bentley A, Klein KU, Kwiecien R, Shi C, Szczyrba M, David M, Markstaller K (2013) An inhaled tumor necrosis factor-alpha-derived TIP peptide improves the pulmonary function in experimental lung injury. Acta Anaesthesiol Scand 57:334–341. 10.1111/aas.12034 23216436 10.1111/aas.12034

[19] Houser A, Baconguis I (2024) Structural insights into subunit-dependent functional regulation in epithelial sodium channels. Structure. 10.1016/j.str.2024.11.013 39667931 10.1016/j.str.2024.11.013 PMC11805665

[20] Huang HL, Hsing HW, Lai TC, Chen YW, Lee TR, Chan HT, Lyu PC, Wu CL, Lu YC, Lin ST, Lin CW, Lai CH, Chang HT, Chou HC, Chan HL (2010) Trypsin-induced proteome alteration during cell subculture in mammalian cells. J Biomed Sci 17:36. 10.1186/1423-0127-17-36 20459778 10.1186/1423-0127-17-36 PMC2873939

[21] Huber R, Krueger B, Diakov A, Korbmacher J, Haerteis S, Einsiedel J, Gmeiner P, Azad AK, Cuppens H, Cassiman JJ, Korbmacher C, Rauh R (2010) Functional characterization of a partial loss-of-function mutation of the epithelial sodium channel (ENaC) associated with atypical cystic fibrosis. Cell Physiol Biochem 25:145–158. 10.1159/000272059 20054153 10.1159/000272059

[22] Hughey RP, Bruns JB, Kinlough CL, Harkleroad KL, Tong Q, Carattino MD, Johnson JP, Stockand JD, Kleyman TR (2004) Epithelial sodium channels are activated by furin-dependent proteolysis. J Biol Chem 279:18111–18114. 10.1074/jbc.C400080200 15007080 10.1074/jbc.C400080200

[23] Hughey RP, Mueller GM, Bruns JB, Kinlough CL, Poland PA, Harkleroad KL, Carattino MD, Kleyman TR (2003) Maturation of the epithelial Na^+^ channel involves proteolytic processing of the alpha- and gamma-subunits. J Biol Chem 278:37073–37082. 10.1074/jbc.M307003200 12871941 10.1074/jbc.M307003200

[24] Ilyaskin AV, Korbmacher C, Diakov A (2021) Inhibition of the epithelial sodium channel (ENaC) by connexin 30 involves stimulation of clathrin-mediated endocytosis. J Biol Chem 296:100404. 10.1016/j.jbc.2021.100404 33577799 10.1016/j.jbc.2021.100404 PMC7973139

[25] Kashlan OB, Boyd CR, Argyropoulos C, Okumura S, Hughey RP, Grabe M, Kleyman TR (2010) Allosteric inhibition of the epithelial Na^+^ channel through peptide binding at peripheral finger and thumb domains. J Biol Chem 285:35216–35223. 10.1074/jbc.M110.167064 20817728 10.1074/jbc.M110.167064 PMC2966135

[26] Kashlan OB, Wang XP, Sheng S, Kleyman TR (2024) Epithelial Na^+^ channels function as extracellular sensors. Compr Physiol 14:1–41. 10.1002/cphy.c230015 39109974 10.1002/cphy.c230015 PMC11309579

[27] Kellenberger S, Schild L (2015) International Union of Basic and Clinical Pharmacology. XCI. structure, function, and pharmacology of acid-sensing ion channels and the epithelial Na^+^ channel. Pharmacol Rev 67:1–35. 10.1124/pr.114.009225 25287517 10.1124/pr.114.009225

[28] Kino-oka M, Taya M (2009) Recent developments in processing systems for cell and tissue cultures toward therapeutic application. J Biosci Bioeng 108:267–276. 10.1016/j.jbiosc.2009.04.007 19716513 10.1016/j.jbiosc.2009.04.007

[29] Kleyman TR, Eaton DC (2020) Regulating ENaC’s gate. Am J Physiol Cell Physiol 318:C150–C162. 10.1152/ajpcell.00418.2019 31721612 10.1152/ajpcell.00418.2019 PMC6985836

[30] Kleyman TR, Kashlan OB, Hughey RP (2018) Epithelial Na^+^ channel regulation by extracellular and intracellular factors. Annu Rev Physiol 80:263–281. 10.1146/annurev-physiol-021317-121143 29120692 10.1146/annurev-physiol-021317-121143 PMC5811403

[31] Lai TY, Cao J, Ou-Yang P, Tsai CY, Lin CW, Chen CC, Tsai MK, Lee CY (2022) Different methods of detaching adherent cells and their effects on the cell surface expression of Fas receptor and Fas ligand. Sci Rep 12:5713. 10.1038/s41598-022-09605-y 35383242 10.1038/s41598-022-09605-y PMC8983651

[32] Lemmens-Gruber R, Tzotzos S (2023) The epithelial sodium channel-an underestimated drug target. Int J Mol Sci 24. 10.3390/ijms24097775 10.3390/ijms24097775 PMC10178586 37175488

[33] Letz B, Ackermann A, Canessa CM, Rossier BC, Korbmacher C (1995) Amiloride-sensitive sodium channels in confluent M-1 mouse cortical collecting duct cells. J Membr Biol 148:127–141. 10.1007/BF00207269 8606362 10.1007/BF00207269

[34] Lu M, Echeverri F, Kalabat D, Laita B, Dahan DS, Smith RD, Xu H, Staszewski L, Yamamoto J, Ling J, Hwang N, Kimmich R, Li P, Patron E, Keung W, Patron A, Moyer BD (2008) Small molecule activator of the human epithelial sodium channel. J Biol Chem 283:11981–11994. 10.1074/jbc.M708001200 18326490 10.1074/jbc.M708001200

[35] Lucas R, Yue Q, Alli A, Duke BJ, Al-Khalili O, Thai TL, Hamacher J, Sridhar S, Lebedyeva I, Su H, Tzotzos S, Fischer B, Gameiro AF, Loose M, Chakraborty T, Shabbir W, Aufy M, Lemmens-Gruber R, Eaton DC, Czikora I (2016) The Lectin-like Domain of TNF Increases ENaC Open Probability through a Novel Site at the Interface between the Second Transmembrane and C-terminal Domains of the alpha-Subunit. J Biol Chem 291:23440–23451. 10.1074/jbc.M116.718163 27645999 10.1074/jbc.M116.718163 PMC5095400

[36] Martin-Malpartida P, Arrastia-Casado S, Farrera-Sinfreu J, Lucas R, Fischer H, Fischer B, Eaton DC, Tzotzos S, Macias MJ (2022) Conformational ensemble of the TNF-derived peptide solnatide in solution. Comput Struct Biotechnol J 20:2082–2090. 10.1016/j.csbj.2022.04.031 35601958 10.1016/j.csbj.2022.04.031 PMC9079168

[37] Masilamani S, Kim GH, Mitchell C, Wade JB, Knepper MA (1999) Aldosterone-mediated regulation of ENaC alpha, beta, and gamma subunit proteins in rat kidney. J Clin Invest 104:R19-23. 10.1172/JCI7840 10510339 10.1172/JCI7840 PMC408561

[38] Mutchler SM, Kirabo A, Kleyman TR (2021) Epithelial sodium channel and salt-sensitive hypertension. Hypertension 77:759–767. 10.1161/HYPERTENSIONAHA.120.14481 33486988 10.1161/HYPERTENSIONAHA.120.14481 PMC7878349

[39] Nesterov V, Bertog M, Canonica J, Hummler E, Coleman R, Welling PA, Korbmacher C (2021) Critical role of the mineralocorticoid receptor in aldosterone-dependent and aldosterone-independent regulation of ENaC in the distal nephron. Am J Physiol Renal Physiol 321:F257–F268. 10.1152/ajprenal.00139.2021 34251271 10.1152/ajprenal.00139.2021 PMC9847332

[40] Nesterov V, Dahlmann A, Bertog M, Korbmacher C (2008) Trypsin can activate the epithelial sodium channel (ENaC) in microdissected mouse distal nephron. Am J Physiol Renal Physiol 295:F1052-1062. 10.1152/ajprenal.00031.2008 18653483 10.1152/ajprenal.00031.2008

[41] Nesterov V, Dahlmann A, Krueger B, Bertog M, Loffing J, Korbmacher C (2012) Aldosterone-dependent and -independent regulation of the epithelial sodium channel (ENaC) in mouse distal nephron. Am J Physiol Renal Physiol 303:F1289-1299. 10.1152/ajprenal.00247.2012 22933298 10.1152/ajprenal.00247.2012

[42] Nesterov V, Krueger B, Bertog M, Dahlmann A, Palmisano R, Korbmacher C (2016) In Liddle syndrome, epithelial sodium channel is hyperactive mainly in the early part of the aldosterone-sensitive distal nephron. Hypertension 67:1256–1262. 10.1161/HYPERTENSIONAHA.115.07061 27170740 10.1161/HYPERTENSIONAHA.115.07061

[43] Noreng S, Bharadwaj A, Posert R, Yoshioka C, Baconguis I (2018) Structure of the human epithelial sodium channel by cryo-electron microscopy. Elife 7. 10.7554/eLife.39340 10.7554/eLife.39340 PMC6197857 30251954

[44] Noreng S, Posert R, Bharadwaj A, Houser A, Baconguis I (2020) Molecular principles of assembly, activation, and inhibition in epithelial sodium channel. Elife 9. 10.7554/eLife.59038 10.7554/eLife.59038 PMC7413742 32729833

[45] Obergrussberger A, Bruggemann A, Goetze TA, Rapedius M, Haarmann C, Rinke I, Becker N, Oka T, Ohtsuki A, Stengel T, Vogel M, Steindl J, Mueller M, Stiehler J, George M, Fertig N (2016) Automated patch clamp meets high-throughput screening: 384 cells recorded in parallel on a planar patch clamp module. J Lab Autom 21:779–793. 10.1177/2211068215623209 26702021 10.1177/2211068215623209

[46] Obergrussberger A, Friis S, Bruggemann A, Fertig N (2021) Automated patch clamp in drug discovery: major breakthroughs and innovation in the last decade. Expert Opin Drug Discov 16:1–5. 10.1080/17460441.2020.1791079 32646308 10.1080/17460441.2020.1791079

[47] Obergrussberger A, Goetze TA, Brinkwirth N, Becker N, Friis S, Rapedius M, Haarmann C, Rinke-Weiss I, Stolzle-Feix S, Bruggemann A, George M, Fertig N (2018) An update on the advancing high-throughput screening techniques for patch clamp-based ion channel screens: implications for drug discovery. Expert Opin Drug Discov 13:269–277. 10.1080/17460441.2018.1428555 29343120 10.1080/17460441.2018.1428555

[48] Obergrussberger A, Rinke-Weiss I, Goetze TA, Rapedius M, Brinkwirth N, Becker N, Rotordam MG, Hutchison L, Madau P, Pau D, Dalrymple D, Braun N, Friis S, Pless SA, Fertig N (2022) The suitability of high throughput automated patch clamp for physiological applications. J Physiol 600:277–297. 10.1113/JP282107 34555195 10.1113/JP282107

[49] Passero CJ, Carattino MD, Kashlan OB, Myerburg MM, Hughey RP, Kleyman TR (2010) Defining an inhibitory domain in the gamma subunit of the epithelial sodium channel. Am J Physiol Renal Physiol 299:F854-861. 10.1152/ajprenal.00316.2010 20630937 10.1152/ajprenal.00316.2010 PMC2957262

[50] Pearce D, Manis AD, Nesterov V, Korbmacher C (2022) Regulation of distal tubule sodium transport: mechanisms and roles in homeostasis and pathophysiology. Pflugers Arch 474:869–884. 10.1007/s00424-022-02732-5 35895103 10.1007/s00424-022-02732-5 PMC9338908

[51] Piercy KT, Donnell RL, Kirkpatrick SS, Mundy BL, Stevens SL, Freeman MB, Goldman MH (2001) Effect of harvesting and sorting on beta-1 integrin in canine microvascular cells. J Surg Res 100:211–216. 10.1006/jsre.2001.6247 11592795 10.1006/jsre.2001.6247

[52] Rapedius M, Obergrussberger A, Humphries ESA, Scholz S, Rinke-Weiss I, Goetze TA, Brinkwirth N, Rotordam MG, Strassmaier T, Randolph A, Friis S, Liutkute A, Seibertz F, Voigt N, Fertig N (2022) There is no F in APC: using physiological fluoride-free solutions for high throughput automated patch clamp experiments. Front Mol Neurosci 15:982316. PMID: 36072300. 10.3389/fnmol.2022.982316 10.3389/fnmol.2022.982316 PMC9443850 36072300

[53] Rauh R, Soell D, Haerteis S, Diakov A, Nesterov V, Krueger B, Sticht H, Korbmacher C (2013) A mutation in the beta-subunit of ENaC identified in a patient with cystic fibrosis-like symptoms has a gain-of-function effect. Am J Physiol Lung Cell Mol Physiol 304:L43-55. 10.1152/ajplung.00093.2012 23087020 10.1152/ajplung.00093.2012

[54] Reichard A, Asosingh K (2019) Best practices for preparing a single cell suspension from solid tissues for flow cytometry. Cytometry A 95:219–226. 10.1002/cyto.a.23690 30523671 10.1002/cyto.a.23690 PMC6375754

[55] Rossier BC (2014) Epithelial sodium channel (ENaC) and the control of blood pressure. Curr Opin Pharmacol 15:33–46. 10.1016/j.coph.2013.11.010 24721652 10.1016/j.coph.2013.11.010

[56] Rotin D, Staub O (2021) Function and regulation of the epithelial Na^+^ channel ENaC. Compr Physiol 11:2017–2045. 10.1002/cphy.c200012 34061979 10.1002/cphy.c200012

[57] Rotordam MG, Obergrussberger A, Brinkwirth N, Takasuna K, Becker N, Horvath A, Goetze TA, Rapedius M, Furukawa H, Hasegawa Y, Oka T, Fertig N, Stoelzle-Feix S (2021) Reliable identification of cardiac conduction abnormalities in drug discovery using automated patch clamp II: best practices for Na 1.5 peak current in a high throughput screening environment. J Pharmacol Toxicol Methods 112:107125. 10.1016/j.vascn.2021.107125 10.1016/j.vascn.2021.107125 34500078

[58] Roush GC, Ernst ME, Kostis JB, Yeasmin S, Sica DA (2016) Dose doubling, relative potency, and dose equivalence of potassium-sparing diuretics affecting blood pressure and serum potassium: systematic review and meta-analyses. J Hypertens 34:11–19. 10.1097/HJH.0000000000000762 26556568 10.1097/HJH.0000000000000762

[59] Ruffieux-Daidie D, Poirot O, Boulkroun S, Verrey F, Kellenberger S, Staub O (2008) Deubiquitylation regulates activation and proteolytic cleavage of ENaC. J Am Soc Nephrol 19:2170–2180. 10.1681/ASN.2007101130 18701608 10.1681/ASN.2007101130 PMC2573013

[60] Shabbir W, Scherbaum-Hazemi P, Tzotzos S, Fischer B, Fischer H, Pietschmann H, Lucas R, Lemmens-Gruber R (2013) Mechanism of action of novel lung edema therapeutic AP301 by activation of the epithelial sodium channel. Mol Pharmacol 84:899–910. 10.1124/mol.113.089409 24077967 10.1124/mol.113.089409 PMC3834145

[61] Shimkets RA, Warnock DG, Bositis CM, Nelson-Williams C, Hansson JH, Schambelan M, Gill JR Jr, Ulick S, Milora RV, Findling JW et al (1994) Liddle’s syndrome: heritable human hypertension caused by mutations in the beta subunit of the epithelial sodium channel. Cell 79:407–414. 10.1016/0092-8674(94)90250-x 7954808 10.1016/0092-8674(94)90250-x

[62] Staudner T, Geiges L, Khamseekaew J, Sure F, Korbmacher C, Ilyaskin AV (2024) Disease-associated missense mutations in the pore loop of polycystin-2 alter its ion channel function in a heterologous expression system. J Biol Chem 300:107574. 10.1016/j.jbc.2024.107574 39009345 10.1016/j.jbc.2024.107574 PMC11630642

[63] Sure F, Afonso S, Essigke D, Schmidt P, Kalo MZ, Nesterov V, Kissler A, Bertog M, Rinke R, Wittmann S, Broeker KAE, Gramberg T, Artunc F, Korbmacher C, Ilyaskin AV (2024) Transmembrane serine protease 2 and proteolytic activation of the epithelial sodium channel in mouse kidney. J Am Soc Nephrol. 10.1681/ASN.0000000521 10.1681/ASN.0000000521 PMC11888964 39441656

[64] Sure F, Bertog M, Afonso S, Diakov A, Rinke R, Madej MG, Wittmann S, Gramberg T, Korbmacher C, Ilyaskin AV (2022) Transmembrane serine protease 2 (TMPRSS2) proteolytically activates the epithelial sodium channel (ENaC) by cleaving the channel’s gamma-subunit. J Biol Chem 298:102004. 10.1016/j.jbc.2022.102004 35504352 10.1016/j.jbc.2022.102004 PMC9163703

[65] Sure F, Einsiedel J, Gmeiner P, Duchstein P, Zahn D, Korbmacher C, Ilyaskin AV (2024) The small molecule activator S3969 stimulates the epithelial sodium channel by interacting with a specific binding pocket in the channel’s beta-subunit. J Biol Chem 300:105785. 10.1016/j.jbc.2024.105785 38401845 10.1016/j.jbc.2024.105785 PMC11065748

[66] Tzotzos S, Fischer B, Fischer H, Pietschmann H, Lucas R, Dupre G, Lemmens-Gruber R, Hazemi P, Prymaka V, Shabbir W (2013) AP301, a synthetic peptide mimicking the lectin-like domain of TNF, enhances amiloride-sensitive Na current in primary dog, pig and rat alveolar type II cells. PulmPharmacol Ther 26:356–363. 10.1016/j.pupt.2012.12.011 10.1016/j.pupt.2012.12.011 PMC3646188 23313096

[67] van Roy F, Berx G (2008) The cell-cell adhesion molecule E-cadherin. Cell Mol Life Sci 65:3756–3788. 10.1007/s00018-008-8281-1 10.1007/s00018-008-8281-1 PMC11131785 18726070

[68] Vassalli JD, Belin D (1987) Amiloride selectively inhibits the urokinase-type plasminogen activator. FEBS Lett 214:187–191. 10.1016/0014-5793(87)80039-x 10.1016/0014-5793(87)80039-x 3106085

[69] Zhang B, Shan H, Li D, Li ZR, Zhu KS, Jiang ZB, Huang MS (2012) Different methods of detaching adherent cells significantly affect the detection of TRAIL receptors. Tumori 98:800–803. 10.1177/030089161209800619 10.1177/030089161209800619 23389369

[70] Zhou Q, Wang D, Liu Y, Yang X, Lucas R, Fischer B (2017) Solnatide demonstrates profoundtherapeutic activity in a rat model of pulmonary edema induced by acute hypobaric hypoxia andexercise. Chest 151:658–667. 10.1016/j.chest.2016.10.030 27815150 10.1016/j.chest.2016.10.030

