# Supplementary material for: Automated patch-clamp recordings for detecting activators and inhibitors of the epithelial sodium channel (ENaC)
Source: Pflugers Arch. 2025 May 8;477(6):857–72. doi: 10.1007/s00424-025-03087-3 (PMC12092551; doi:10.1007/s00424-025-03087-3)
Supplement: Supplementary file 1 — Supplementary file1 (DOCX 2141 KB) [file 424_2025_3087_MOESM1_ESM.docx]

# **Western Blot Raw Data (full uncropped blots)**

*Supplementary data to Figure 1a.*

### **α-ENaC**

| **1** | Molecular weight markers (half of the lane) |
| --- | --- |
| **2** | Control HEK293 cells |
| **3** | ENaC-HEK293 cells |
| **4** | Molecular weight markers |

|  | **1** | **2** | **3** | **4** |  |
| --- | --- | --- | --- | --- | --- |
| 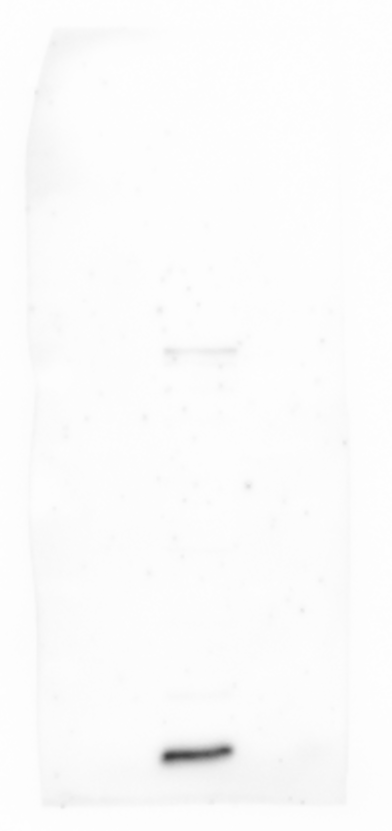 | | | | | |

### **β-ENaC**

| **1** | Molecular weight markers (half of the lane) |
| --- | --- |
| **2** | Control HEK293 cells |
| **3** | ENaC-HEK293 cells |
| **4** | Molecular weight markers (half of the lane) |

|  | **1** | **2** | **3** | **4** |  |
| --- | --- | --- | --- | --- | --- |
| 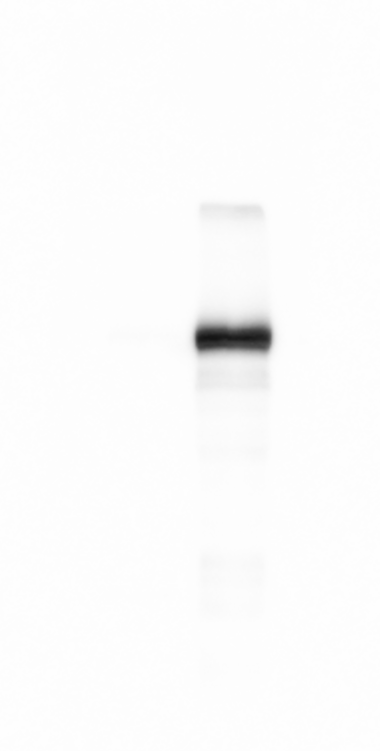 | | | | | |

### **γ-ENaC / β-actin**

| **1** | Molecular weight markers |  |
| --- | --- | --- |
| **2** | Control HEK293 cells | Cell surface protein |
| **3** | ENaC-HEK293 cells | Cell surface protein |
| **4** | Molecular weight markers |  |
| **5** | Control HEK293 cells | Intracellular protein |
| **6** | ENaC-HEK293 cells | Intracellular protein |
| **7** | Molecular weight markers |  |
| **8** | Control HEK293 cells | Intracellular protein |
| **9** | ENaC-HEK293 cells | Intracellular protein |

*Note that lanes 5 and 8, as well as lanes 6 and 9 are technical replicates. Only lanes 2,3 and 8,9 are shown in Figure 1a.*

**γ-ENaC**

|  | **1** | **2** | **3** | **4** | **5** | **6** | **7** | **8** | **9** |  |
| --- | --- | --- | --- | --- | --- | --- | --- | --- | --- | --- |
| 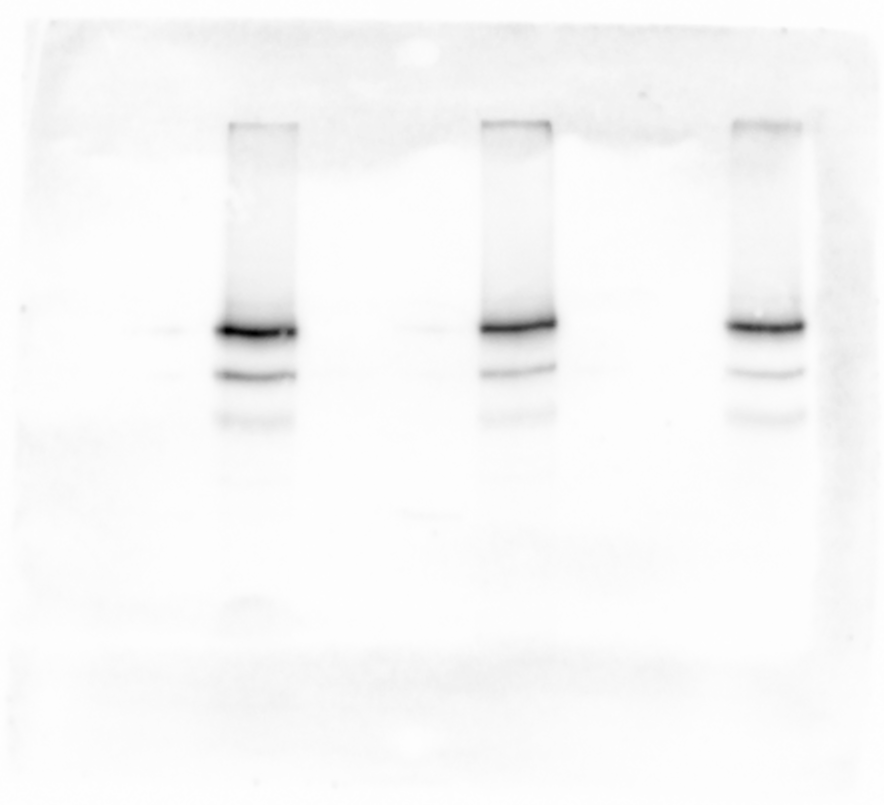 | | | | | | | | | | |

**β-actin**

|  | **1** | **2** | **3** | **4** | **5** | **6** | **7** | **8** | **9** |  |
| --- | --- | --- | --- | --- | --- | --- | --- | --- | --- | --- |
| 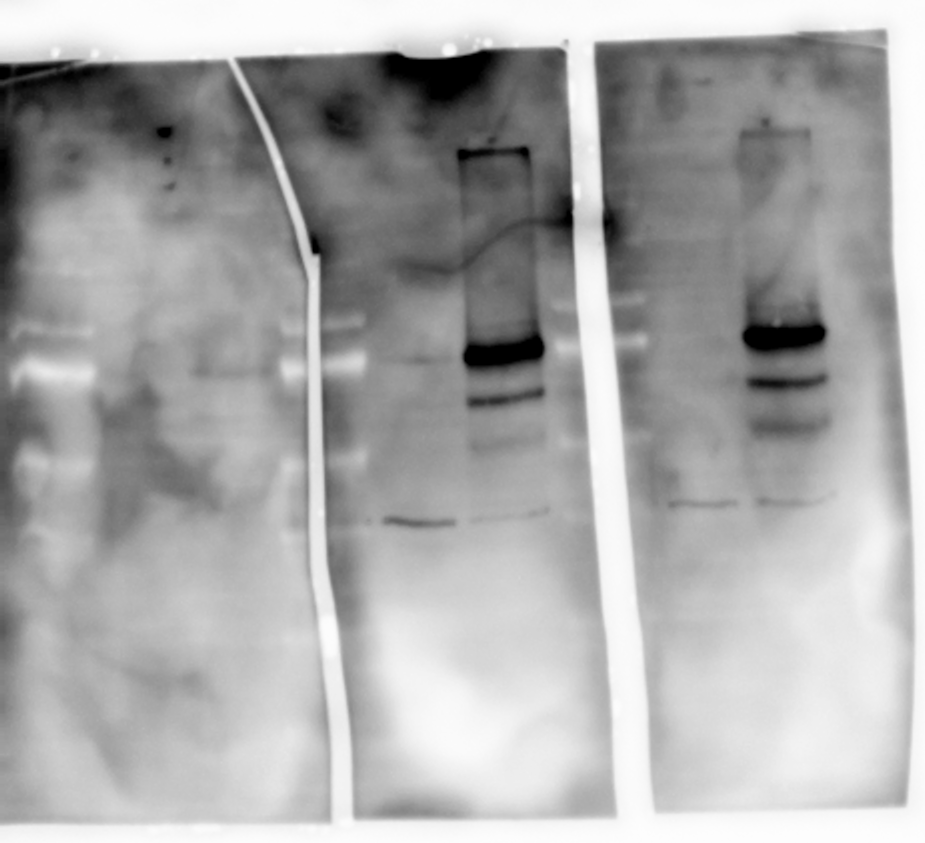 | | | | | | | | | | |
