# Supplementary material for: Automated patch-clamp recordings for detecting activators and inhibitors of the epithelial sodium channel (ENaC)
Source: Pflugers Arch. 2025 May 8;477(6):857–72. doi: 10.1007/s00424-025-03087-3 (PMC12092551; doi:10.1007/s00424-025-03087-3)
Supplement: Supplementary file 2 — Supplementary file2 (DOCX 993 KB) [file 424_2025_3087_MOESM2_ESM.docx]

**Automated patch-clamp recordings for detecting activators and inhibitors
of the epithelial sodium channel (ENaC)**

*- Supplementary Information -*

Florian Sure^1^, Markus Rapedius^2^, Alexei Diakov^1^, Marko Bertog^1^, Alison Obergrussberger^2^,
Niels Fertig^2^, Christoph Korbmacher^1^, Alexandr V. Ilyaskin^1^

| ^1^ | Friedrich-Alexander-Universität Erlangen-Nürnberg, Institute of Cellular and Molecular Physiology, Erlangen, Germany |
| --- | --- |
| ^2^ | Nanion Technologies GmbH, Munich, Germany |
|  |  |
| Table of Contents **Supplemental Figure S1** Enzymatic cell detachment reagents lead to proteolytic activation of ENaC heterologously expressed in *Xenopus laevis* oocytes  **Supplemental Figure S2** Similar APC recordings as shown in Fig. 4 were performed in an additional batch of ENaC-HEK293 cells and confirmed the suitability of this method to detect stimulatory and inhibitory effects of known ENaC modulators on ENaC currents | |


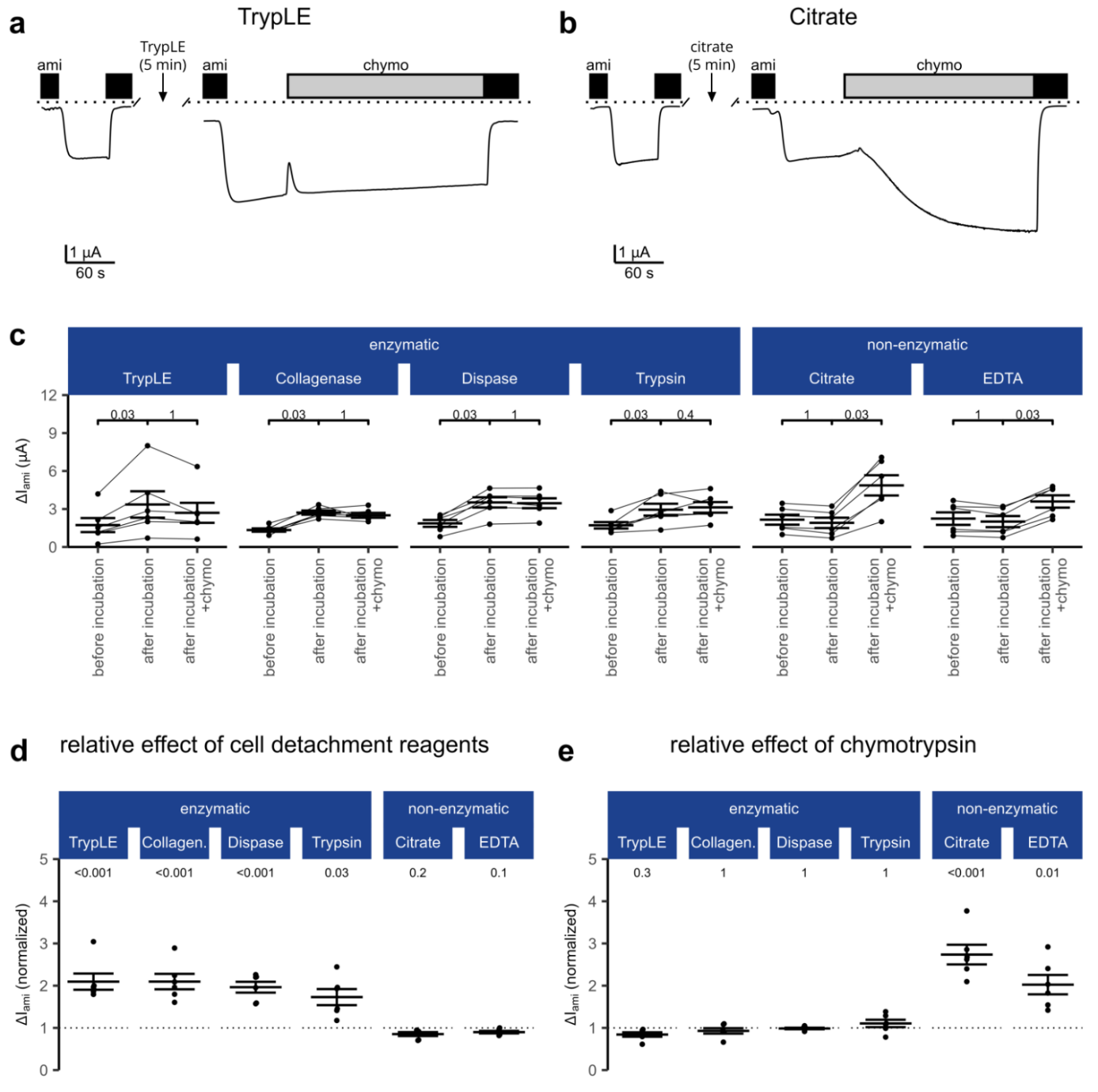


**Supplemental Fig. S1 Enzymatic cell detachment reagents lead to proteolytic activation of ENaC heterologously expressed in *Xenopus laevis* oocytes**

| **(a, b)** | Representative whole-cell current traces obtained at a holding potential of −60 mV are shown for an oocyte injected with cRNA encoding for human αβγ-ENaC (0.03 ng/subunit/oocyte) before and after incubation with TrypLE (*a*) or sodium citrate (*b*). Amiloride (ami, 2 µM) and chymotrypsin (chymo, 2 µg/ml) were present in the bath solution as indicated by black and grey bars, respectively. An arrow marks a 5-minute incubation time in the respective reagent, as indicated, followed by 1 minute of washout with the standard bath solution containing amiloride (2 µM). During this incubation time, oocytes were unclamped to prevent sodium overloading. |
| --- | --- |
| **(c)** | ENaC-mediated amiloride-sensitive whole-cell currents (ΔI_ami_) were determined from similar experiments as shown in (*a,* TrypLE) and (*b,* Citrate) and from experiments in which other cell detachment reagents were used, as indicated. Values were determined by subtracting the current level reached in the absence of amiloride before incubation in the respective detachment reagent (before incubation), or after incubation in the detachment reagent (after incubation), or at the end of chymotrypsin application (after incubation + chymo) from the current level in the presence of amiloride. Lines connect data points obtained in each individual oocyte. Mean ± SEM and individual data points are shown. Paired one-sided two-sample Wilcoxon Signed Rank test (n=6) with Bonferroni correction for multiple testing. |
| **(d, e)** | Relative effects of the cell detachment reagents (*d*) and chymotrypsin (*e*) on ΔI_ami_ calculated from data shown in (*c*). The dotted line indicates a relative effect of one (no effect). Mean ± SEM and individual datapoints are shown. Two-sided one-sample t-test with Bonferroni correction for multiple testing of logarithmic values. |


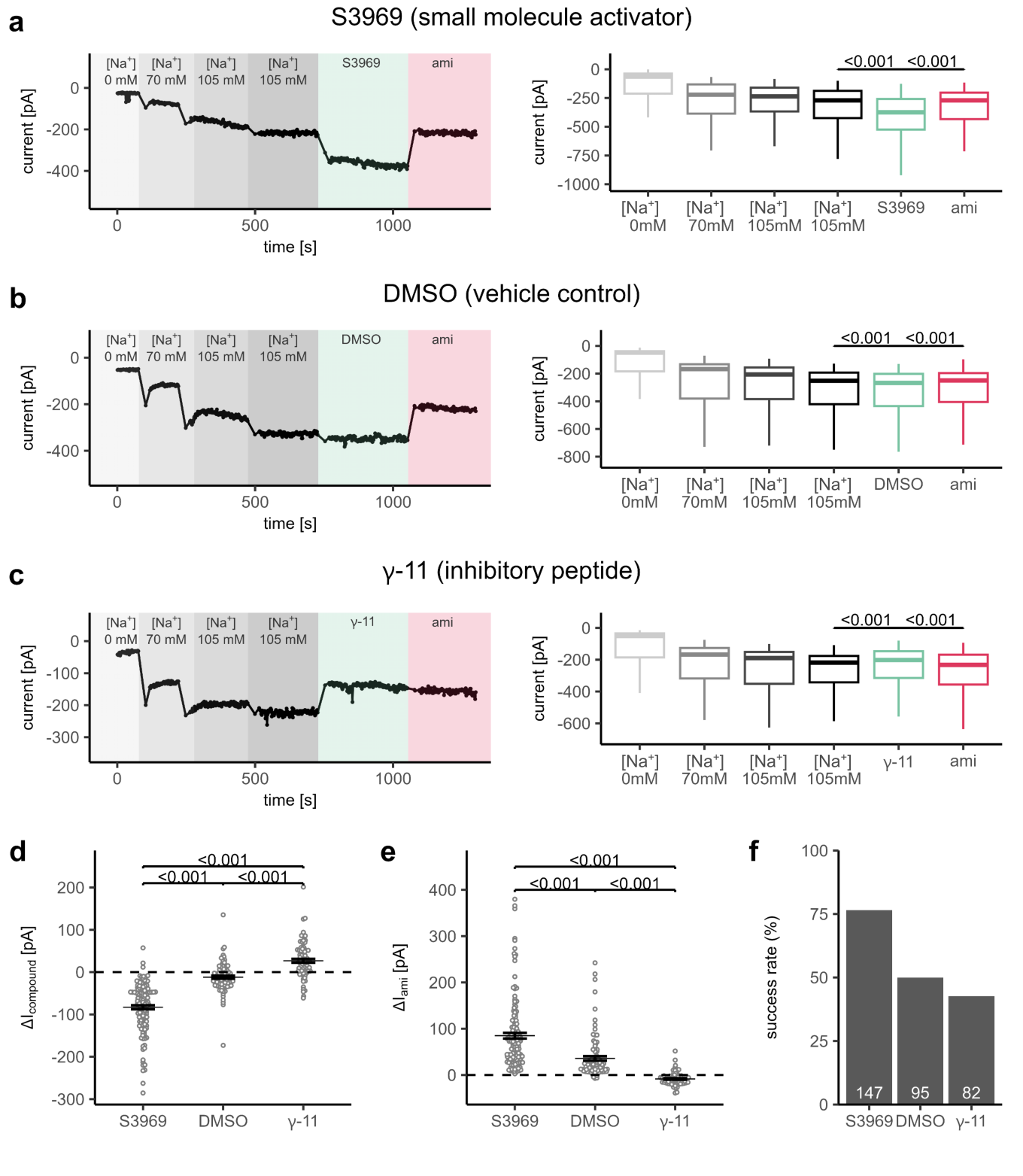


**Supplemental Fig. S2 Similar APC recordings as shown in Fig. 4 were performed in an additional batch of ENaC-HEK293 cells and confirmed the suitability of this method to detect stimulatory and inhibitory effects of known ENaC modulators on ENaC currents**

| **(a-c)** | *Left panels:* Representative APC recordings obtained using a multi-hole chip (4 holes per well, 4x S-Type; Nanion, product no.: 22 2401). Background colours and labels indicate bath solutions. Measurements started with a similar solution exchange protocol as described in Figure 2a, b. Green indicates subsequent application of the small-molecule ENaC-activator S3969 (*a*; S3969, 10 µM), the vehicle-control DMSO (*b;* DMSO, 0.01% v/v), or the synthetic ENaC-inhibiting 11-mer peptide (*c;* γ-11, 10 µM). Violet indicates application of amiloride at the end of the recording (ami, 10 µM).  *Right panels:* Summary data from similar experiments as in the corresponding *left panels*. Current levels were determined at the end of each phase, as described in Figure 2a, b. Two-sided, paired Student’s *t*-test (*a:* n=147, *b:* n=95, *c:* n=82). |  |
| --- | --- | --- |
| **(d, e)** | Absolute effect of S3969, DMSO, or γ-11 (*d*, ΔI_compound_), or subsequently applied amiloride (*e*, ΔI_ami_) on currents calculated from data shown in (*a-c*). Values were obtained as described in Figure 2c, d. The dotted line indicates an absolute effect of zero (no effect). Mean ± SEM and individual datapoints are shown. Two-sided Student’s *t*-test. |  |
| **(f)** | The ratio of successful recordings to all recordings in the respective experimental group, calculated as described in Figure 4e. Numbers in white within each bar represent the number of successful recordings per group. |  |
